# Acknowledgment to the Reviewers of *Genes* in 2022

**DOI:** 10.3390/genes14020238

**Published:** 2023-01-17

**Authors:** Genes Office Editorial

**Affiliations:** MDPI AG, St. Alban-Anlage 66, 4052 Basel, Switzerland

High-quality academic publishing is built on rigorous peer review. *Genes* was able to uphold its high standards for published papers due to the outstanding efforts of our reviewers. Thanks to the efforts of our reviewers in 2022, the median time to first decision was 17 days and the median time to publication was 38 days. Regardless of whether the articles they examined were ultimately published, the editors would like to express their appreciation and thank the following reviewers for the time and dedication that they have shown *Genes*:
A. Garcia-RuizLeonid BystrykhA. K. M. Mahmudul HuqueLeonor García-GómezA. M. Abu AhmedLetizia Li PianiA. N. M. Mamun-Or-RashidLetizia PalombaA. Pratap KumarLev PorokhovnikA.K.M. Aminul IslamLewis Vibul HunAaron E. LinLeyland FraserAaron F. SeversonLi FengAaron GrossLi LiAaron LeichtyLi YizhouAarti SharmaLi YuAasim MajeedLiang ZhangAbani Kanta NaikLida FuentesAbbu ZaidLidan YeAbd Elaleim ElsayedLidia Gonzalez-QueredaAbdelghafar Abu-ElsaoudLidia Hanna MarkiewiczAbdelkarim MahdhiLidia Lasecka-DykesAbdenour KheloufiLidia StrigariAbdolkarim ZareiLidia Szulc-DąbrowskaAbdolrazagh Hashemi ShahrakiLies LahousseAbdul Hafiz Ab MajidLiezhao LiuAbdul SattarLifei LiAbdullahi Adekilekun JimohLige TongguAbdullahi MuhammadLihong LiAbdulmojeed YakubuLi-Jun HuangAbhijit RathLilach SoreqAbhik GhoshLiliana AscioneAbhinav AdhikariLiliane N. TandziAbhinava MishraLiming ShenAbhishek ChandraLin AnAbhishek KulkarniLin ChenAbhishekh BhandawatLin LiuAboElenain MansourLin SongAbolfazl BahramiLin ZhangAbraham Escobar-GutiérrezLin ZhaoAbril GijsbersLinchen HeAbubakr H. MossaLinda BellucciAda ColluraLinda HouhamdiAdam AbiedLinda PetersAdam BurtonLinda PetijováAdam CawleyLindsay M. LaFaveAdam J. ShapiroLindsay T. MichaloviczAdam WilkinsLing ChenAdelaida AvinoLing SunAdeline SnehaLing YinAdi PerelmanLing ZhuAdil AkkouchLingling MiaoAdil KhanLingling ZhengAdina BrahaLingxiao HeAditya LahiriLinhua WangAditya Reddy KolliLinjie WangAdnan Shami ShahLinlin GuoAdrian Canizalez-RomanLinna AnAdrián CasanovaLinyan MengAdrián GarridoLinyuan MaAdrian SmedowskiLiron RozenkrantzAdriana DerejkoLisa B NamerowAdriana Fernanda Kuckartz VizueteLisa Privette VinnedgeAdriana FodorLisbeth TranebjærgAdriana GeisingerLisha MouAdriana GrigorașLiudmila V. SpirinaAdriana Mariel GentileLivio VitielloAdriana VinhasLivius V. D'UscioAdrianna VlachosLjiljana BrbaklićAdriano CoelhoLjubica OdakAdriano PereiraLlorenç Fernández-CollAdriano TagliabracciLluis MontoliuAdwoa Asante-PokuLokesh KumarAfreen HussainLong ZhangAfroz AlamLopa MishraAfsal KolloliLorena ButinarAftab BashirLorena Joga-ElviraAftab YaseenLorena UrbanelliAfzal HussainLorenzo Felipe Sanchez-TeyerAgat Leonska-DuniecLorenzo NeviAgata Wawrzkiewicz-JałowieckaLorenzo TalaricoAgathe BourgmayerLoretta DorstynÁgnes TantosLori S. SullivanAgnieszka BieniekLou MetherellAgnieszka GachLouis-Charles BélandAgnieszka JeleńLouise Mary BurmeisterAgnieszka Lecka-AmbroziakLu LiAgnieszka Madej-PilarczykLu WangAgnieszka MostowskaLuca AmbrosioAgnieszka Siomek-GoreckaLuca BelloAgnieszka SkarzyńskaLuca BragaAgre PaterneLuca GiacomelliAgustín ArandaLuca LovrecicAhamad AlqudahLuca PuzziAhlam M. AlhusainiLucas BoatwrightAhmad A. OmarLucia Maria ProcopciucAhmad Jawad SabirLucia Vieira HoffmannAhmad Sofiman OthmanLuciana Bolsoni LourençoAhmed AbdelghanyLuciana CaenazzoAhmed AteyaLuciana Diniz RolaAhmed ElbestawyLuciana MusanteAhmed El-HossaryLuciano ColangeloAhmed ElmassryLucie Biehler-GomezAi My LuongLucien BovetAida Andrades ValtueñaLucio BertarioAida PetcaLucky Poh Wah GohAihua ShaLudmiła Grzybowska-SzatkowskaAimee TaylorLuigi CaputiAimy SebastianLuigi XodoAinhoa AgorretaLuigia D. De FalcoAinhoa Robles-MezcuaLuis Andrés López FernándezAinong ShiLuis Andreu CaravacaAisha SyedaLuis Augusto Eijy NagaiAishwarya GurumurthyLuis Eduardo Servín GarcidueñasAiti VizziniLuis Exequiel IbarraAitor SerresLuis Fernando Jacinto-AlemánAjay OblaLuis RamírezAjay ThakurLuís SilvaAjda MoškričLuisa BernardinelliAjinkya KawaleLuisa CarbogninAjit GhoshLuisa GarofaloAjit SharmaLuísa M.P. ValenteAjith AshokanLuisa NardiniAkalabya BissoyiLuisa PolitanoAkhilanand ChaurasiaLukáš KratochvílAkhtar AkhtarLukas VargaAkiko KozakiŁukasz KajtochAkiko SuzukiLukasz PrzybylAkio OishiŁukasz WalasAkira IshikawaŁukasz WojtylaAkira IwaseLuke HessonAkriti SrivastavaLunda ShenAlaeldein M. AbudabosLu-Sheng HsiehAlain RamanantsoanirinaLu-Sheng XinAlain VincentLuyen VuAlakananda DasLuz Berenice López-HernándezAlan B. RoseLynne M. BirdAlan Le MoanLyubov E. SalnikovaAlan WuLyubov GetmantsevaAlan YuLyudmila F. GulyaevaAlanna StrongLyudmila KhrabrovaAlberto Barreras-SerranoM. Anwar IqbalAlberto García-RedondoM. Beatriz Durán AlonsoAlberto Marin-SanguinoM. R. MohammadabadiAlberto MavilioM. Teresa TejedorAlberto Molares VilaM. Z. JankovicAldo DonizettiMaad ShatnawiAlejandro SaettoneMaarouf AdilAleksandra JancevskaMaarten KamermansAleksandra Sałagacka-KubiakMac GardnerAleksandra SuchaneckaMaciej KurpiszAleksei N. SuminMaciej SkorackiAleš BlincMaciej TarnowskiAles CveklMadelyn GillentineAlessandra AntonucciMadhuja SamaddarAlessandra De FeoMadhumala K. SadanandappaAlessandra P. PrincivalleMadhusmita PanigrahyAlessandro AchilliMagdalena Gayà-VidalAlessandro BenedettoMagdalena KrocAlessandro D’AmuriMagdalena LisAlessandro GiulianiMagdalena M. BusAlessandro LoglioMagdalena M. MaslonAlessandro LucianiMagdalena MroczekAlessandro OttaianoMagdalena OrzechowskaAlessandro PigoniMagdalena RudzinskaAlessandro VittoriMagdalena SkarzynskaAlessia ArcaroMagdalena StasiakAlessia ColosimoMagdalena Zatoń-DobrowolskaAlessia Di CostanzoMagdalena ŻurawekAlessia FaggianMagdalene J. SeilerAlessia GambadoroMaged M. A. FoudaAlessio BellatoMagella M. NeveuAlessio IannucciMahara ValverdeAlessio SquassinaMahesh NeupaneAlex TroitskyMahesh Raj NepalAlexander A. IshchenkoMahipal KesawatAlexander AwgulewitschMahipal Singh KesawatAlexander BerezinMahmoud MadkourAlexander EreskovskyMahmoud MagdyAlexander GraphodatskyMahtab NourbakhshAlexander Hesselberg LøvestadMaikel Castellano-PozoAlexander KanapinMainak MustafiAlexander KrivoruchkoMaíra RodriguesAlexander LykovMaja BoczkowskaAlexander SuhMaja GregićAlexander TamalunasMaja MatulicAlexander V. ProninMaja ŠrutAlexander Vasilievich KarpukhinMajid VahedAlexandra AbdelmanovaMaksim AksenovAlexandra MartuMalcolm D. SchugAlexandra PauloMałgorzata AdamczukAlexandra RibaritsMałgorzata Kotula-BalakAlexandra RosaMałgorzata Natonek-Wi´sniewskaAlexandra ZakharenkoMałgorzata OżgoAlexandre SmirnovMalgorzata PilotAlexandros PapachristodoulouMalgorzata Tyszka-CzocharaAlexei SlepzofMalik SajidAlexey M. BurdennyyMamdouh M. A. Awad-AllahAlexey MorgounovMan P. HuynhAlexey MoseykoManal FawzyAlexey V. PindyurinManas KinraAlfredo GueraManas KotepuiAlfredo PulvirentiManasi TamhankarAlhamzawi RahimMandi GandelmanAli BadawyManee M. ManeeAli Chenari BouketManfred KlaasAli El-RaghiMangesh DudheAli JavadmaneshManish CharanAli MaghsoudiManish ManraiAli Mohammad WaryhaManish TripathiAli SakhdariManish Vijaya KumarAli SalamiManjeet SinghAlia TelliManoj KumarAlice TraversaManolis LadoukakisAlice Verticchio VercellinMansour LamjedAlicja BorońManu PrasadAlicja PacholewskaManuel Aira VieiraAlina BabenkoManuel Alejandro MerloAlina TruţăManuel BelliAlireza ZarebidokiManuel Sanchez-DiazAlison G. Ossip-DrahosManuela ColosimoAlison Starr-MossMao MaoAlla P. MironenkoMar InfanteAllan BayatMara GyöngyvérAllen Sam TitusMarc A. BakerAlma D. Alarcon-RojoMarc Anton FuessingerAlmerinda AgrelliMarc BlondelAlok ArunMarc GhesquiereAlok Ranjan SahuMarc HeuermannAlvaro Del RealMarc LlavaneraÁlvaro Plaza ReyesMarc T. FacciottiAlysia CoxMarc TatarAmal A. Al-ShargabiMarc Van HulleAmalio Santacruz VarelaMarc Woodbury-SmithAman Ullah KhanMarcel HanischAmancio CarneroMarcel Matei DudaAmanda KowalczykMarcel MéchaliAmaniel KefleyesusMarcel SchmidtAmany MohamedMarcel TawkAmar SinghMarcela RodrigueroAmardeep Singh VirdiMarcella MassiminiAmaro SacoMarcella ZollinoAmel Gritli-LindeMarcello MezzasalmaAmelia CasamassimiMarcello MocciaAmelia Portillo LópezMarcello TagliaviaAmets Sáenz PeñaMarcelo CorreiaAminallah TahmasebiMarcelo F. SimonAmir Amiri-YektaMarcelo R. Sánchez-VillagraAmir AriffMarcelo Tigre MouraAmir MahboubiMarcia ManterolaAmir SagiMarcia SaraivaAmir ShemiraniMarcin MichalakAmit DhingraMarcin NicośAmit KumarMarcin OzarowskiAmjad HussainMarcin R. LenerAmjad KhanMarcin RatajewskiAmmar EbrahimiMarcin RóżewiczAmnon KorenMárcio F. SimãoAn Phu Tran NguyenMárcio Fernando Ribeiro ResendeAn ZhuMarco CerranoAna C. GonçalvesMarco Di StefanoAna Gisela ReyesMarco DolciAna Isabel López SeséMarco GalaverniAna López-MalvarMarco GerdolAna Luísa De Sousa-CoelhoMarco GrodzkiAna Luisa Garcia OliveiraMarco JoseAna MagalhãesMarco NutiAna NikolicMarco SavareseAna Paez-GarciaMarco TecillaAna Paula SantosMarcus CoratAna ReisMarek D. KoterAna Silvia A. M. T. MouraMarek KiliszekAnalía del Valle GarneroMarek LubośnyAnamaria NecşuleaMarek MuriasAnand LalliMarek PieszkaAnanda Virgínia De AguiarMarek RuchałaAnandrao Ashok PatilMarek ŚwitońskiAnanthapur VenkateshwariMargarida Barcelo-SerraAnastas GospodinovMargarita A. SazonovaAnastasia KazantsevaMargarita LamprouAnastasiya V. SnezhkinaMargarita M. MarquesAnat Yaskolka MeirMargarita MeerAnbarasu KannanMargherita CerroneAnca Angela SimionescuMari Anne PhilipsAnca Lucau-DanilaMaria A. ArvioAnders MalmströmMaria Antonietta RagusaAnderson Ferreira Da CunhaMaria ArnedoAndi FebrisiantosaMaria BallesterAndrás HidasMaria BovaAndras KomaromyMaria C. AlonsoAndrás MolnárMaria Cristina Romero-RodríguezAndraz DovnikMaría Del Rayo SánchezAndré A. SantosMaria Do Carmo PeluzioAndre LechelMaría Dolores Llobat BordesAndre MegarbaneMaría Esther EstebanAndré SchallerMaria GerakariAndre TavaresMaria Giuseppina MianoAndrea BeccioliniMaria GomezAndrea C. RinaldiMaria HernandezAndrea CarraMaría Inés PigozziAndrea DekanicMaría J. MartínAndrea GelemanovicMaría Jesús Alvarez CuberoAndrea Henriques PonsMaria Jorge Geraldes CamposAndrea L. GropmanMaría José Bautista-LlamasAndrea RandoMaria José Ladera-CarmonaAndrea ScharfMaria LongeriAndrea TelatinMaria Lucia Carneiro VieiraAndrea UrbaniMaria LucibelloAndrea VaagsMaria Luisa DettoriAndrea ViscaMaria Malane Magalhães MunizAndrea VisioniMaria Montiel-TroyaAndreas BrodehlMaría Moreno-del ÁlamoAndreas ChiocchettiMaria N. TutukinaAndreas R. JaneckeMaria NilssonAndreas WeberMaria NishevaAndreia BrandaoMaria OczkowiczAndrej ZupanMaria Paola MogaveroAndrés FloresMaria PapasavvaAndrés J. CortésMaria Patrizia StoppelliAndres López-QuinteroMaria Sabater-MolinaAndrew C. DellMaria ShadrinaAndrew G. ClarkMaria SharakhovaAndrew Li YimMaria SollerAndrew MacnabMaria SpletterAndrew MallettMaria Suarez DiezAndrew MurphyMaria Susana LopesAndrew OzgaMaría Teresa Cubo SánchezAndrew PoveyMaria Teresa VietriAndrew R. BondMaria TokamaniAndrew SchurkoMaria TsiarliAndrew ShedlockMaria Valeria EspositoAndrey A. SinjushinMaria ValievaAndrey CherstvyMariaelena RepiciAndrey GrigorievMaría-José ArgenteAndrey MironovMariamena ArbitrioAndrey SinjushinMarian BresticAndrey V. FrolovMariana Pereira AntoniassiAndrey ZamyatninMariana R. BottonAndrey ZhelankinMariangela ManciniAndrzej DybusMariann HarangiAndrzej L. ChamieniaMarianna DománAndrzej TeisseyreMarianna E. WeenerAndrzej ZieleniakMariano Andres Loza-CollAnela IvanovaMariano Francesco CaratozzoloAnella SavianoMariano IntrieriAneta BalcerczykMariarita BrancaccioÁngel Aledo-SerranoMaribel Forero-CastroAngela RoggeroMarica CarielloAngela RosenbohmMarie BaucherAngela StarkweatherMarie SisslerAngela TeaniMarie-Louise KampmannAngelica MiglioliMarije MeuwissenAngélica Saraí Jiménez-OsorioMarijn Van Der VeldeAngelika KrólMarika PellegriniAngelo De PaolisMarilena BolcatoAngelo SiricoMarina Durán-AguilarAngelos KalitzeosMarina GomzikovaAnida Maria BabtanMarina NaoumkinaAnil A. ChuturgoonMarina NonicAnil AnnamneediMarina PiscopoAnil ChekuriMarina S. AscunceAnil KumarMarina SelionovaAnil SinghMarinela BostanAnil TharappelMario GorenjakAnil VermaMario ZuritaAnindya GangulyMariona TerradasAnirban ChakrabortyMarius ZahanAnisa RibaniMariusz SkowronskiAnita M. SheteMariz VainzofAnita QuintanaMariza G. MorgadoAnja MottokMark A. SteinAnja WeiseMark AntrobusAnkita PunethaMark W. RussellAnkush AuradkarMarko TrajkovskiAnmin LeiMarkus ReischlAnn StockerMaróti-Agóts ÁkosAnna AspesiMarta BassittaAnna BarbaroMarta DiepenbroekAnna CieślińskaMarta GerasymchukAnna Di MatteoMarta HidalgoAnna GaertnerMarta Kaczmarek-RyśAnna Jakubska-BusseMarta LlopAnna KoseniukMarta OlszewskaAnna KouznetsovaMarta SarkozyAnna KusienickaMarta Stankiewicz-KosylAnna LetkoMarta Żebrowska-NawrockaAnna LinderholmMartha LegorretaAnna M. GrabowskaMartin BartasAnna Maria D'ErchiaMartin FeulnerAnna MichalczykMartin John DedicoatAnna MigdałMartin KatzAnna NowickaMartin KnytlAnna Paola CapraMartín Mata RosasAnna PapadopoulouMartin RichardsAnna Rita RossiMartin SabolAnna StefanskaMartin SchmidtAnna V. LomakinaMartin SztachoAnna VaskuMartin Tristani-FirouziAnna Vittoria CarluccioMartin VingronAnna WawruszakMartin ZiegerAnna Wierzbicka-WośMartin ZofallAnna Wołoszyn-DurkiewiczMartina CaiazzaAnna Zalewska-JanowskaMartina CardoniAnna Zielak-SteciwkoMartina MiluchováAnnabel S. BerthonMartina SandonáAnna-Sophia KiangMartina TorricelliAnne BassettMartine CrosetAnne Elisabeth MercierMarvin MillerAnne M. RochtusMarwa A. A. FayedAnne SchänzerMarwa MatboliAnne SimmenrothMary B. FaroneAnne-Marie GalowMary C. MajAnoop AlexMaryam DaneshpourAnshika GoenkaMaryam FarzanehAnshul TiwariMaryam JavedAnssi L. VuorinenMaryam KeshavarziAnte IvankovićMary-Louise RisherAnthony MillarMarzena Wińska-KrysiakAnthony MukwayaMarziliano NicolaAnthony WangMasafumi MurataniAntimo Di MaroMasahiro KanedaAntje BanningMasahito YamagataAntoine FagesMasanao MiwaAntoine PegatMasaru TanakaAnton KomarMasashi MizuguchiAnton TyurinMasato SuzukiAntonella MinelliMasatsugu HatakeyamaAntonia BrunoMassimiliano ChettaAntonieta Chávez-GonzálezMassimiliano CoccaAntonietta FrancoMassimiliano Di VittorioAntonina ShumakovaMassimo GiovannottiAntónio AmorimMassimo La RosaAntonio BarbatoMassimo RomaniAntonio BellastellaMatas JakubauskasAntonio BrunoMatěj PánekAntonio Campos-CaroMathew AlexanderAntonio ColantuoniMathias ToftAntonio Francesco Maria GiulianoMathieu CoppeyAntonio G. SolimandoMatija RijavecAntonio Giovanni SolimandoMatilda NewtonAntonio GiovinoMatt PowellAntonio IeniMatteo BozzoAntonio Juan PazosMatteo CarpiAntonio Llombart-CussacMatteo FabbriAntonio MassaroMatteo FlorisAntonio PalazzoMatteo MartinaAntonio PizzutiMatthew E. R. ButchbachAntonio SantanielloMatthew G. JohnsonAntonio SermekMatthew J. AndersonAntonio Victor Campos CoelhoMatthew S. MacauleyAntonios ZambounisMatthew SudermanAntt Htet WaiMatthias CarlAnuj KumarMatthias DrostenAnukana BhattacharjeeMattia ConteAnupam MukherjeeMattia PelizzolaAnupama SharmaMauricio BustosAnurag MishraMauricio Comas-GarciaAnurag MisraMauricio CuelloAnurag ParanjapeMaurizio MargaglioneAnush AghajanyanMaurizio RomanoApoorv TiwariMauro NirchioApostolos PapachristosMauro RaposoAqeel AhmadMaxime PichonAraz R. KiviMaximilian MichelArbi GuetatMaxwell E. R. ShaferArchana PrabaharMayukh GuhaAri MeersonMazahar MoinAriadni MentiMazène HochaneArianna LatiniMazharul IslamArijita SarkarMcLain AdamAris KaltsasMd. Abdullah Al BariAristidis StavroulopoulosMd. Abul Hassan SameeAristotelis C. PapageorgiouMd. Atikur RahmanArkadiusz KajdaszMd. Jahirul IslamArkadiusz PrzybyszMd. Saiful IslamArkadiusz UrbańskiMd. Shahid SarwarArkom ChaiwongkotMediha TrabelsiArman RahimmiMeetali SinghArnab GuptaMegan B. MurrayArnab PalMegan PovelonesArnab SenMeghan MoranArnaud HeckerMehanathan MuthamilarasanArnaud LombardMehdi MontazerArnone JamesMehdi RezaeiArnulfo Hernan Nava-ZavalaMehdi ShakibaeiArsen ArakelyanMehdi SoltaniArsen DotsevMehdi Younesi-HamzekhanluArshad AlhasnawiMehmet BozkurtArtak HeboyanMehmet BülbülArthitaya Kawee-aiMehmet GültasArtur BurzyńskiMehmet Hadi AydinArtur PałaszMehmet Ilyas CosacakArtur Schumacher-SchuhMehmet KaracaArturo Sánchez-GonzálezMehmet KizilaslanArun Kumar Hanumana Gouda PatilMehmet Ulaş ÇinarArun Kumar JoshiMei YangArun RenganathanMeiji Soe AungArun SrivastavaMei-Kuang ChenAruna PalMeiyun FanArunachalam MuthuramanMelanie J. PercyArunika DasMeng MaoArwa ShahinMeng WuAsad AliMenghuan TangAshish JainMeng-Leong HowAshkan ZandiMengxi ShenAshok MohantyMeran Keshawa EdiriweeraAshraf Al AshhabMerita XhetaniAshutosh KumarMert MestanogluAshutosh PandeyMerve KasapAshutosh RaiMesut KeserAsif NadeemMian Abdur Rehman ArifAsim Kumar BepariMian Faisal NazirAsim MuhammadMichael A. TrakselisAsma AyazMichael AradAsmuni Mohd IkmalMichael BeckerAsuman GedikbasiMichael BrentAsunción LagoMichael CobleAtefeh SabouriMichael CunninghamAtiah H. AlmalkiMichael E. DavisAtif AdnanMichael G. KempAtiyeh M AbdallahMichael GreenacreAtsuo IidaMichael HeckerAtsuo KikuchiMichael HofreiterAtsushi IshiharaMichael J. CurtisAtsushi KatoMichael James GilhooleyAttila FarsangMichael Jordan RowleyAttila KissMichael KristensenAttila ZsolnaiMichael McDermottAttiq Ur RehmanMichael McGowenAtul PranayMichael R. HowittAudrey GuenicheMichael RyanAurel Popa-WagnerMichael S. AdamowiczAurora StorlazziMichael SwansonAustin LarsonMichael TellierAvik ChoudhuriMichael TemplinAvinash MishraMichael W. MarshallAvinash PremrajMichael WellingtonAvinash V. KarpeMichael WempeAwdhesh MishraMichaela A. BotiAyako OkuzakiMichail RovatsosAyca KocaagaMichailidis MichailAyman Gamal Fawzy EL NagarMichał BulcAzadeh Akhavan RoofigarMichal GáborAzhahianambi PalavesamMichal Jan MarkuszewskiAzza MohamedMichał KulusBabar HussainMichal R. SchweigerBabatunde RajiMichał SzopińskiBabette GüntherMichal VostrýBac Viet LeMichalis KaramouzisBahattin TanyolacMichel WassefBahman KhahaniMichela BattistelliBakytzhan BekmanovMichela ClericiBalaji SadhasivamMichela LandoniBalakuntalam KasinathMichela RossiBalázs EgyedMichele A. BattleBalazs VargaMichele Di CosolaBálint NagyMichele ManganelliBang LiuMichele ZaccaiBao LiuMichel-Edwar MickaelBaofeng SuMichelle BarthetBaoyin ZhaoMichiko ShigyoBaptiste BidonMietje GermonpréBarbara Eleni RosatoMiguel Angel Alcántara-OrtigozaBarbara GaravagliaMiguel Ángel Hernández-OñateBarbara Kloeckener-GruissemMiguel Ángel Reyes-LópezBarbara MarquesMiguel Leiva-BrondoBarbara MiziakMihir SarkarBarbara PawłowskaMikhail AlexeyevBarbara Van AschMikhail DubininBaris OzyerMikhail GantsevichBarry M. WillardsonMikhail KryuchkovBart De GeestMikhail PyatnitskiyBartłomiej BudnyMiklós I. TóthBartlomiej KrazinskiMilagros Sánchez MayorBartosz PłachnoMilan Kumar LalBasharat AliMiloš MacholánBashayer R. Al-MubarakMiluše VozdováBayodé Roméo AdégbitèMilyausha KaskinovaBayram ToramanMin Jung KwunBayrem JemmaliMin Kyu KimBeata BakeraMinakshi GandhiBeáta HubkováMine KoruyucuBeata NowakMinerva Codruta BadescuBeata PyrzakMing LiBéatrice TurcqMing Li WangBeatriz M. Navarro-DomínguezMingfu WuBeatriz Martínez-PovedaMing-Jing HwangBeatriz PuisacMing-Kun HsiehBee K. TanMingli XuBee Kang TanMing-Sheng TengBehnam Asgari LajayerMingsheng YangBela BalintMingzhi LiaoBelaghihalli N. GnaneshMingzhuo LiBen GutierrezMin-Hyeon ParkBen MansMinjie ZhangBen WielstraMinjun YangBenedetta PerroneMinkyu ParkBeniamin Oskar GrabarekMinoo RassoulzadeganBenjamin BrigantMintu MeenaBenjamin N. SacksMir Ajab KhanBenjamin RocheMirabela-Smaranda AlecsaBenoit BizimunguMirai AzumaBenoît LacombeMiranda Mladinić PejatovićBenoit MiottoMireia UrangaBernard BinetruyMirela CordeaBernard De MassyMirela LozićBernard DujonMireya Burgos-HernándezBernardo BertoniMiriam DomowiczBernardo ChavesMiriam ElbrachtBernardo Dias PereiraMiriam J. SmithBernat CrosasMiriam PotronyBernd LenzMiriam Saiz-RodrigezBernd WissingerMirjana Babić LekoBetrouni NacimMirko ManchiaBhakti BasuMiron SopićBhola Shankar PradhanMiroslav PlohlBhupendra Singh PanwarMirosław AndrusiewiczBhupendra VermaMiroslawa DabertBianyun YuMirza HasanuzzamanBibekananda KarMisbahuddin M. RafeeqBibha ChoudharyMishra Vikash ChandraBibo JiangMitchell M HollandBichun LIMitsue NishiyamaBidisha RoyMitsuhiro TakenoBijay BeheraMizuho Sato-DahlmanBin GuMobashir MohammadBin LiangMobina AlemiBin LiuMoeti Oriel TaioeBin WuMoghoofei MohsenBin ZhuMohamed A. El-EsawiBinod KumarMohamed AliBiragyn AryaMohamed E. Abd El-HackBiswajit PadhyMohamed ElmonemBjarne UddMohamed ElshalBjörn VoßMohamed El-SodaBlake SweeneyMohamed GadBo HuMohamed H. Al-AgamyBo YanMohamed KaramBo ZhangMohammad AshfaqBo ZhouMohammad Atikur RahmanBobrek KamilaMohammad Bagher HassanpouraghdamBogdan I. GerashchenkoMohammad Fahad UllahBogdan SmykMohammad Faujul KabirBogna Grygiel-GórniakMohammad H. AbukhalilBojan ŽunarMohammad Israil AnsariBojjibabu ChidipiMohammad KamranBoleslaw KarwowskiMohammad Kazem SouriBoopathi SubramaniyanMohammad Mahdi ForghanifardBorong ShaoMohammad Reza AshrafzadehBostan NazishMohammad Reza Khodaei ArdakaniBoubacar Amadou KountchéMohammad SouriBowen SongMohammad Tahir SiddiquiBoyang LiMohammad ZulkifliBožena Ćurko-CofekMohammed A. RohaimBrad A. AmendtMohammed AliBrad A. FrekingMohammed AourachBrandon J. WaltersMohammed GagaouaBrandon McKinneyMohammed RhaziBranislav ŠilerMohan GuptaBranko JovcicMohan HaleyurgirisettyBraxton D. MitchellMohanned Naif AlhussienBreckpot JeroenMohanta Tapan KumarBrendan ReidMohd Saleem DarBrian FoutyMohit K. MidhaBrian K. FoutchMohsen HesamiBrian MortonMohsen MohammadBrian VerrelliMojtaba KordrostamiBrigitte GalliotMolly NicodemusBrijesh SinghMonali NandyMazumdarBrinda BalasubramanianMónica ArakakiBrînduşa Alina PetreMonica CappettaBruna CarbasMónica VieiraBrunno Rocha LevoneMonika K. Grudzinska-PechhackerBruno FerretteMonika PechhackerBruno FossoMonika Stefaniuk-SzmukierBruno GomesMonther T. SadderBruno Oliveira Da Silva DuranMorais PauloBrutch NinaMoran GershoniBryan T. HackfortMorten GrauslundBryn WebbMoses AyoolaBudsaraporn NgampanyaMostafa K. SarmastBurak KaracaörenMotoko UnokiBurcu Balcı-HaytaMozheh ZamiriBurkhard BergerMpho MafaBushra IjazMridul JohariByoung-Il BaeMuhamad Noor Alfarizal KamarudinByung Soo KimMuhamad Thohar ArifinC. ScapoliMuhammad Abu Bakar SaddiqueÇağrı ErginMuhammad AfzalCai ZongMuhammad Amjad NawazCaifei ZhangMuhammad AsifCaihong DongMuhammad Asif NaveedCaihong WeiMuhammad Bilal AzmiCaitriona M. McEvoyMuhammad ImranCameron SnellMuhammad IqbalCandan HizelMuhammad KhanCao YangMuhammad KhurramCara TimpaniMuhammad Muaaz AslamCarina TörnMuhammad Mubashar ZafarCarina VisserMuhammad NaeemCarine ArnoniMuhammad Nasir Khan KhattakCarine TisnéMuhammad Ramzan KhanCarl WeinerMuhammad SajjadCarla Maria CalòMuhammad Shahid IqbalCarla PollastroMuhammad SohailCarlo NobileMuhammad Sohail ZafarCarlo SalaMuhammad Tarek Abdel GhafarCarlos Alberto FernandesMuhammad UmairCarlos D. BruqueMuhammad Wahab AmjadCarlos EscuderoMuhammad ZafarCarlos Fernando Prada QuirogaaMuhammad-Shoaib AkhtarCarlos I. ArbizuMuhammed A. AcikgozCarlos Iglesias PastranaMuhammet Ali GundesliCarlos Perez-PlasenciaMuhsin ElmasCarlos Torres-TorresMukesh KumarCarmela PaolilloMukesh MeenaCarmelo BiondoMukesh P. YadavCarmen BerasainMukund SrinivasanCarmen CadillaMuntazir MushtaqCarmen Guillén-PonceMurali MamidiCarmen Rojas-MartínezMurray PattersonCarol MasonMusa KavasCarole A. Samango‐SprouseMustafa AkçelíkCarolina BizamaMustafa CesurCarolina BonillaMustafa HititCarolina ConstantinMustapha Cherkaoui MalkiCarolina Isabel MiñoMuthu ArumugamCaroline Theresa SeebauerMuthu SathishCarolyn M. KlingeMuthu ThiruvengadamCarolyn M. MacicaMuthusubramanian VenkateshwaranCarrie WelchN. RusenovaCasco Robles Martin MiguelNA AbdullahCatalina SantiagoNa SongCatarina C. PachecoNadia MucciCathy BessudoNadine MoubayedCatia ScassellatiNady GolestanehCecilia DemergassoNagah ArafatCedrik Tekendo-NgongangNagla Abdel KarimCelal BulgayNaiba NabievaCelia Marisa Rizzatti-BarbosaNajeeb UllahCélia NogueiraNajmaldin SakiCéline BonNakao KuboCéline KosterNalini DhingraCelso Mendes-JuniorNam HuynhCemal ÜnNan SongCengiz OzalpNándor LiptákCesar Cortez-RomeroNaoki KabeyaCésar López-CamarilloNaoki YamamotoCesar Marcial Escobedo-BonillaNaoko TakezakiCesare DanesinoNaomi A. PorretCeyda KasaviNaomi P. FriedmanChak-Sum HoNaoto HanzawaChan WangNaoyuki KataokaChandra Mohan SinghNarayanasamy BadrinathChandra Sekhar BathulaNarongrit MuangmaiChandrika PereraNarottam AchyaChang LiNaruo NikohChang LuNasim Ahmad YasinChangcheng WuNasser GhanemChangdong WangNatale MussoChang-il HwangNatalia DementevaChanglong WenNatalia Garcia-GiraltChantal Me E. TallaksenNatalia Hernandez-PachecoChantal PileggiNatalia KasicaChao ZhangNatalia MalaraChaoqun HuangNatalia Martínez-GilChaoxing LiuNatalia MilerCharalampos ThomasNatalia ParamonovaCharikleia KelaidiNatalia V. BelosludtsevaCharlene HanlonNatalia Wawrusiewicz-KurylonekCharles Nathan HancockNatalia Yanguas-CasásCharuta AgasheNatalia ZinovievaChava Kimchi-SarfatyNatalie J. ForsdickChen ChuNatalie L. ListerChen DingNataliya K. KovtonyukChen LiNatalya A. ShnayderChen MoNatalya GlushkovaChen Siang NgNatapol PornputtapongCheng ChangNataša Marčun VardaChenghao ChenNatasha Andressa Nogueira JorgeCheng-Ming ChuongNathalie GontierChenguang FanNathalie OulhenChengyi SongNathan B. SutterChengyu YuNathan ScudderChet RamNathaniel H. RobinCheuk Lun LiuNaveen DuhanChia-Liang WuNaveen Kumar C. GowdaChiara GabelliniNavid AbedpoorChiara PlatellaNavid Ghavi Hossein-ZadehChiara RizziNavin KumarChiara VillaNavin Kumar DevarajChieh Lee WongNavneet MatharuChien-Feng LiNavraj Kaur SaraoChih-Yang WangNayun KimChina NaganoNazar A. ShapovalChinedu Charles NwaforNazarii KobyliakChing-Yi ChenNazia NazarChi-Nien ChenNeal PeacheyChinmay JaniNeal T. DittmerChinna Venkata Subbaiah KadiamNeda AničićChinnamani PrasannakumarNedenia Bonvino StafuzzaChintan KapadiaNeelam KumariChirag GuptaNeeracha SanchavanakitChiranjeevi PadalaNeeraj KumariChi-Ting HorngNeeti Sanan MishraChitong RaoNeha A. ThakreChitra RawatNeha DesaiChiu-Jung HuangNeha NandaChiung-Ting WuNehal F. HassibChiyoko MachidaNejc UmekChloe M. StantonNelly MohamedChong LiNemanja RajčevićChongkui SunNematzadeh SajjadChongmei DongNenad DelićChrista F. HonakerNeonila V. KononenkoChristian BarbatoNetty SalindehoChristian Griñán-FerréNeus BaenaChristian PosberghNeus Font-PorteriasChristian SchlöttererNguyen Minh DucChristian SiadjeuNhien Thi NguyenChristian StockmannNiada StefaniaChristina RamirezNiaz AliChristine BoneNicholas DelihasChristine H. FoyerNicholas F. ParrishChristine L. MummeryNicholas OwenChristine Le SignorNicholas PeakeChristine LerouxNichole Danielle AllredChristine MilcarekNico NagelkerkeChristine NardiniNicola MondanelliChristine RondaninoNicola MontemurroChristoph BudjanNicola PuglieseChristophe DesterkeNicolae GicaChristophe EscudéNicolai Van OersChristophe HanoNicolas LeuenbergerChristophe VernyNicolas TremblayChristopher A. WassifNicole CrownChristopher CollinsNicole Van DeenenChristopher EhrhardtNicole WeisschuhChristopher GrunseichNicoleta RaduChristopher I. JonesNicoletta StaropoliChristopher JohnsonNicolò Maria BuffiChristopher M. HollenbeckNida AslamChristopher PhillipsNidhi DwivediChristopher WardellNidhi SharmaChristos K. KontosNiels FockeChristos NoutsosNiels MorlingChristyraj Johnson Retnaraj Samuel SelvanNiina BhattaraiChrysanthi VoyiatzakiNikita Khromov BorisovChuen-Fu LinNiklas TysklindChukwuemeka George O. Anene-NzeluNiko NjiricChung-Lin LeeNikola ŠtokovićChung-Ta LeeNikolai BorisjukChun-Hong ChenNikolai FriesenChunjiang ZhaoNikolaos MachairasChunlin WangNikolas DovrolisChun-Song YangNikolaus B. BinderChunyang BaoNikolay MehterovChunying LiNikoletta NagyChun-Yu LinNikoletta PsathaChunzhang YangNilanjan ChakrabortyCiesla MaciejNilanjana BanerjeeCigdem AkNilva FurlanCindy KörnerNina MoravčíkováCinzia CiccacciNiraj TripathiCiprian TomuleasaNiroz Abu-Saleh ZoabiCiro IsidoroNishant ChakravortyCitlali Fonseca-GarcíaNishtha NayyarClaire Jean-QuartierNiteshkumar AgarwalClaire Racaud-SultanNitika KurauClaire VandiedonckNitin AmdareClaire WadeNitin KhandelwalClairton Marcolongo-PereiraNitish KumarClara PereiraNitu NigamClarissa Fantin CavarsanNityanand SrivastavaClaude BesmondNkulu Rolly KabangeClaudia AltamuraNneji Lotanna MicahClaudia FalliniNoam D BeckmannCláudia FarinhaNoam JacobClaudia HagedornNobuhiko OkamotoClaudia ManzlNobuko OhmidoCláudia PereiraNoor Al-Huda Ali A. H. SaeedClaudia PiombinoNoor Baity SaidiClaudia RicciNOOR NABILAH TALIK SISINClaudia SissiNoreen F. RossiClaudia TodaroNoreen ZahraClaudia Z. HanNoriko TakegaharaClaudio GrazianoNouf LaqtomClaudio MichelettoNoureddine HamamouchClaudio TomaNuria M. RomeroClaus BørstingNuria P. Torres-AguilaClaus VoglNycolas Levy-PereiraClévia RossetOana Paula PopaClizia VillanoOctavian Tudorel OlaruColm O'RourkeOdile LecompteConcepción De Hita CantalejoØivind BerghConcetta SaocaOksana PogoryelovaCongjun LiOlaia Martinez IglesiasCong-Jun LiOleg DenisenkoConnor BrownOleg FedorchenkoConstantino Macías GarciaOleg RevaConstanza FelizianiOleg Sergeevich AlexandrovConsuelo Rojas IdrogoOlga A. AleynovaCord DrögemüllerOlga BureninaCornel BaltaOlga DolgovaCornelia MirceaOlga ErmakovaCorrado RomanoOlga FedorenkoCosmas NathanailidesOlga HausCostanza Maria CristianiOlga LuzinaCraig A. StockmeierOlga RechkoblitCraig WildingOlga S. FedorovaCristian BonviciniOlga ShatokhinaCristiane KochiOlga ShchaginaCristina CozziOlga SysoevaCristina FlorioOlga V. KochenovaCristina MazzaccaraOlimpia LaiCristina MesquitaOliver J. WatkeysCristina MorsianiOliver KrsticCristina NadaluttiOlivera Mitrović AjtićCristina PolitiOlivier Da InesCristina RiobelloOlivier StaubCristina Rivera-PicónOluwadamilola Faith ShallieCristina SegoviaOm Prakash MeenaCristobal G. D. Dos RemediosOmar Farookh PinjariCrystal MarrugantiOmnia Adel BadrCzarnecki JakubOndrej BonczekDa SunOnur YilmazDacia Dalla LiberaOphélie LebrasseurDae Kwan KoOrestis NousiasDafne Campigli Di GiammartinoOrietta PansarasaDagmara KabzińskaOrish Ebere OrisakweDailu GuanOrsolya Eszter MolnárDainis RungisOsbourne QuayeDaisuke MikiOscar CortesDajun DengOscar Hernández-HernándezDalia MedhatOscar J. TejedaDamiano PiovesanOsman ErkmenDamien DestouchesOtilia-Elena FrasinariuDamien LedererOu ShengDaming ZhuOyetola Olusegun OyebanjiDan DistelÖzgür KasapçopurDan LiOzlem BulbulDan Traian IonescuPablo C. GuerreroDan XuPablo LibradoDana DlouháPablo Luna-NevarezDana Lucia StanculeanuPablo MoraDanai FimereliPablo PulidoDandan YangPablo Thomas-DupontDangwei ZhouPäivi PeltomäkiDanial JahantighPakeerathan KandiahDaniel BernerPalaniyandi KaruppaiyaDaniel BruschiPalavalasa SravyaDaniel Del Valle-MoralesPalok AichDaniel DemarquePamela TozzoDaniel G. MulcahyPan ZhangDaniel García SoutoPanagiotis AthanassiouDaniel GrinbergPanagiotis TheofilisDaniel LundinPankaj AhluwaliaDaniel MeltersPankaj ChaturvediDaniel NolanPankaj KumarDaniel Oruño-SahagunPankaj SharmaDaniel P. PotaczekPaola GrammaticoDaniel P. ZalewskiPaola OstanoDaniel Pierre PetitPaola QuaresimaDaniel PolasikPaola RinchettiDaniel RamsköldPaola RizzoDaniel Romaus-SanjurjoPaolo AbondioDaniel VanekPaolo Armando GagliardiDaniel W. BellottPaolo FagoneDaniel W. NixonPaolo FontanaDaniel YorkPaolo PastorinoDaniela DamianiPaolo RadiceDaniela Elena SerbanPaolo RiccioDaniela IannazzoPaolo SilacciDaniela PalmaParaskevi GoggolidouDaniela PiancatelliParasvi S. PatelDaniela S. IancuPardis NajafiDaniele GuadagnoloParker Lillian D.Daniella DwirParna SahaDanielle Majoor-KrakauerParul VarmaDanilo Trabudo Do AmaralPascal EscherDanique BeijerPascale QuignonDanon Clemes CardosoPasquale De LucaDanuta Babula-SkowrońskaPasquale RaiaDanuta Cembrowska-LechPatrice BourgeoisDanyi LuPatricia Agudelo-RomeroDarae KangPatricia EscandónDaria ShishkovaPatrick J. RichDarin ZertiPatrick JayDario BalestraPatrick Vourc'hDario SiniscalcoPatrizia MalaspinaDario TrapaniPattayampadam Ramakrishnan ShidhiDarius KavaliauskasPaul A. DawsonDariusz KucharczykPaul B. SiegelDarrell L. DinwiddiePaul Charles WhitfordDarren MinierPaul DawsonDaša StupicaPaul GardDasharathraj ShettyPaul HeathDavid A. BenfieldPaul OtyamaDavid A. RayPaul PotterDavid A. WeisblatPaul QuaxDavid AbbottPaul R. MarkDavid Alvarez-PoncePaul S. KruszkaDavid AreshidzePaul T. ThevenotDavid BaldingPaula DoboszDavid BeversdorfPaulina MertowskaDavid ChitayatPauline PriadkaDavid ConversiPauline RomanetDavid CoreyPaulo LinharesDavid CourtneyPaulo Magno Martins DouradoDavid CouvinPavan KumarDavid DoraPavel A. ZykinDavid FerrierPavel LaktionovDavid HaymerPavel SeemanDavid HiggsPawan Kumar RaghavDavid HunterPaweł KonieczkaDavid K. OrrenPaweł KordowitzkiDavid LiberlesPawel MilczarskiDavid LivingstonPayal GangulyDavid M. DonnellPayam SoltanzadehDavid Macaya-SanzPearl Chun ChangDavid MeltonPedro Gonzalez-MenendezDavid Parada DominguezPedro J. AlcoleaDavid Pessanha SiqueiraPedro LebreDavid Sewodor GaikpaPedro Manonelles MarquetaDavid StaceyPedro Martínez-GómezDavid Vicente-ZurdoPei-Chun LiaoDavide PradellaPeiguo YuanDavor NestićPei-Shan HouDawei LiPeitao LüDawei ZhouPenelope AnguileraDawid P. GrzelaPeng GaoDe Jesus Rojas WilfredoPeng ShaoDebabrata DasPeng ZhaoDebasish Kumar DeyPengfei DongDebojit BosePenghao WangDébora Dummer MeiraPenka PetrovaDeborah CharlesworthPenny J. BeuningDeborah J. GoodPentti TienariDeborah RundPer Harald JonsonDeclan P. McKernanPeramaiyan RajendranDeepak KumarPervin Elvan TokgunDeepak SharmaPetar T. TodorovDeepak SinghPeter B. BreslinDeepak VermaPeter Christopher KonturekDeepanjan PaulPeter De KnijffDeependra Kumar SinghPeter DevileeDeepesh GuptaPeter DurdikDeepika PrasadPeter GillDeepika RaiPeter IgazDeirdre DonnellyPeter Ivanov HristovDe-Li ShiPeter J. Koch KochDenis BeliaevPeter JohnstonDenis Copilaş-CiocianuPeter LuntDenis J. HeadonPeter OttDenise HaslingerPeter PelkaDennis McNevinPeter PikoDennis SimpsonPeter PushkoDerek BussanPeter SchielenDerek MorrisPéter TátraiDerek WilkinsonPeter Van HasseltDespoina KalfakakouPetr HanákDespoina ManousakiPetr ŠkorpíkDevesh PantPetr StastnyDevin StauffPetra De GraafDewan Md SumsuzzmanPetra JanovskaDezso ModosPetra KoraćDezsö ModosPetra PennekampDezső MódosPhilip J. HastingsDhamodharan UmapathyPhilip J. YoungDhananjay PandeyPhilippe De MazancourtDhanarajan RajakumarPhilippe HupéDharmendra Kumar YadavPhilippe LeprohonDhaval PatelPhu-Tri TranDhivya ArasappanPier Paolo PiccalugaDi ChenPierfrancesco VisaggiDiaa MoneimPierluigi ScaliaDiaga DioufPiero CossuDiana Cristina Pérez-IbavePierre MoffattDiana Ovejero CrespoPierre RoubertouxDianchang ZhangPierre Simon GarciaDiego A. Esquivel-HernandezPieter AnnaertDiego César Batista MarianoPieter Hermanus MyburghDiego CortezPietro AlanoDiego ForniPietro CrispinoDiego G. OgandoPietro ScicchitanoDiego IngrossoPilar Gomez-GarreDiego Salazar-TortosaPing ChenDiego VerganiPing HeDijana DjureinovicPing SuDik Van GentPingkun ZhouDilip PantheePinky AgarwalDillon MintoffPiotr JanickiDimitri AvramopoulosPiotr OgrodowiczDimitrina MitevaPiotr SzymczykDimitrios LoukovitisPiyush BaindaraDimitrios PanagopoulosPo-Jen HsiaoDimitry GordeninPolly CampbellDimitry SchepetovPonnulakshmi RajagopalDina Mansour Dina Mansour AlyPooja MandkeDingze MangPoonam C. SinghDiogo C. Cabral-de-MelloPrabakaran NagarajanDipak Kumar SahooPrabhakaran SoundararajanDisha SharmaPraddep KumarDivakar SelvarajPradeep CingaramDivya AilPradeep KumarDivya GopinathPradeep Kumar SinghDivya MishraPradip KarmakarDivya SharmaPrafull SalviDivya SinhaPragyesh DhungelDivya VimalPrajish IyerDiyan LiPraopilas PhakdeedindanDmitri FilatovPrasad ParchuriDmitri KramerovPrasanna K. R. AlluDmitrii ShekPrasanthi KogantiDmitriy LajusPrashant MisraDmitry KostyushevPrashant RajbhandariDmitry VerbenkoPrashant VikramDoaa H. ZineldeenPrashanth PrabhuDoaa IbrahimPrashanth SuravajhalaDomenico LioPrasoon AgarwalDomenico MarsonPrathima Perumal ThirugnanasambandamDomenico PignonePraval KhanalDominik DłuskiPraveen F. CherukuriDominika Idziak-HelmckePraveen Kumar Raj KumarDominique S. MichaudPravin KaldhoneDomiziano TarantinoPrecious N. SangoDonald J. ColganPremanand TiwariDonald SetoPrimož KotnikDonatella GambiniPriscilla TngDonato ColangeloPriyanka DhakateDong Gui HuPriyanka GhoshDong HanPriyanka MishraDong MengPriyatansh GurhaDong XinProf Paulo Ricardo ZenDong YangProsanto Kumar ChowdhuryDongdong ZhangProttoy HasanDong-Hyun ShinPui Ying LamDongjing LiuPurushoth EthirajDongliang YuPushpa Raj JoshiDongming LiPushpa RaoDongsheng HuQiang FanDongsheng HuangQiang HuangDong-Xiu XueQiang LiuDongyi BaiQiang ZhangDora Aguín-PomboQianjun ZhaoDorian SargentQiantao ZhengDorota Fopp-BayatQiao XuDorota LechniakQikui WuDorota WłogaQimin HaiDouglas AdamoskiQin WangDouglas BishopQing YangDouglas Silva DominguesQingjiong ZhangDragana MatekaloQingming FangDragana ProticQingyu ZhouDragica SelakovicQinqin PuDror SharonQiong HuDrozdstoj St. StoyanovQiuling LiDubravko ŠkorputQiuwang ZhangDuc Ha ChuQiuyue LiuDuc-Thanh NguyenQuaid HussainDuilio SilvaQuan ZouDuncan TaylorQuanhu ShengDurairaj SekarQuantao MaDusanka Savic-PavicevicQuanxi SunDushyant MishraQuek Zheng Bin RandolphDuy Ngoc DoQuentin C.B. CronkDylan GlubbQuentin ThommenDylan MoliniéQuoc Thang PhamDzmitry ShcharbinQurban AliE. Scott SillsRachel ForrestEbru AtaslarRachel HoustonEbru Karakaya-BilenRachel Ravago-GotancoEbtihal AlshabibRada KazakovaEdivaldo Herculano Correa De OliveiraRadek IndraEdmundo Bonilla GonzálezRadek MalikEdmundo Lozoya-GloriaRadek ProchazkaEdo D’AgaroRadha RamadeviEduard PetitpierreRadhia M'kacherEduardo Arellano-RodrigoRadka SymonovaEduardo Arroyo PardoRadka VáclavíkováEduardo D. MunaizRadomir SavićEduardo Garcia-RicoRadosław ChaberEduardo PimentaRadovan KasardaEduardo SequerraRadu Liviu SumalanEdwin Barrios-VillaRadu MiricaEdwin LasonderRafael De AlmeidaEdyta MakuchRafael Fernández-MartínEfstratios-Stylianos PyrgelisRafael GalupaEhsan AhmadpourRafael KretschmerEhsan RazmaraRafael Plá Matielo LemosEigil KjeldsenRafael Salto-GonzalezEirik SøvikRafael Sanjuan-CerveroEisa TahmasbpourRafael SchroederEiso HiyamaRafael Velázquez-CruzEitan RubinRafał ŚwiechowskiEkaphan KraichakRaffaele Dello IoioEkaterina A. BelousovaRaffaele GarramoneEkaterina A. SemenovaRafiullah RafiullahEkaterina BalaianRaghav KaliaEkaterina MelnikovaRaghavendra YadavalliEkaterina N. TolmachevaRaghvendra DubeyElad LaxRagunathan DevendranEladio VelascoRahmah Al-QthaninElaine OstranderRahul BhowmickElaine SoonRaili KauppinenElcio LealRaimundo Nonato Braga LôboEleanore V. O'NeilRaja Singh PaulrajElena A. AndreevaRajani RaiElena BottaRajeev AuroraElena De FeliceRajeev MehlaElena DrosopoulouRajendran SathishrajElena Gallo MacfarlaneRajesh PandeyElena Garayzábal-HeinzeRajesh ParsanathanElena Iulia IorguRajiv Kumar JhaElena L. GrigorenkoRajiv SahElena Maria ElliRajneesh JaswalElena MartynovaRajneesh PaliwalElena O. VidyaginaRalf BundschuhElena PilliRalf WellingerElena R. SchiffRalf-Erik WellingerElena RossiRalitsa BalkanskaElena S. BelykhRalph FingerhutElena ShakhtshneiderRam SagarElena SommarivaRamadan ArafaElena SticchiRamanagouda BhojappaElena TarcaRambod AbiriElena TovbisRamces Falfan-ValenciaElena ValeevaRamcharan Singh AngomElena ZuriagaRamesh GomezEleni AnastasiadouRamesh Raj PuriEleonora LoiRamesha PapannaEleuterio A. Sánchez-RomeroRami ElshazliElham Karimi-SalesRami MustafaElham PashaeiRami S. NajjarEliana NutricatiRamin SaravaniEliana Saul Furquim Werneck AbdelhayRaminderjit KaurElias TraboulsiRamiro López-MedranoEliecer CotoRamiro Néstor CurtiElina Yankova-TsvetkovaRamona LattaoElisa De FrancoRamya BillurElisa LazzariRandall Felix D'SouzaElisabet JohanssonRandall MaplesElisabeth EhlerRandall MazzarinoElisabetta GazzerroRandi HagermanElisabetta TabolacciRaphael CataneElise LebigotRaquel AndradeElise SchieckRaquel EchavarriaElizabeth McCreadyRashid HussainElizabeth Petri HenskeRashid MirElizeu Fagundes CarvalhoRashmi RashmiEllen KapiteijnRatnakar DeoleElmira MohandesanRavendra P. ChauhanElőd Ernő NagyRavi Chakra TuragaEloisa ArbustiniRavi Kumar GandhamElsayed MansourRavi MuralElsherbiny A. ElsherbinyRavi V. MuralElsherbiny ElsherbinyRavikumar PatelEltyeb AbdelwahidRavindra ThakkarElżbieta Jarocka-CyrtaRaymond CespuglioElżbieta Lodyga-ChruscinskaRaymond WangEman A. RabieRaymundo Rosas-QuijanoEmanuel VamanuRayner Gonzalez PrendesEmanuela SalzanoRazeghian-Jahromi ImanEmanuela Valeria VolpiRăzvan-Ionuț PopescuEmanuela ViggianoRebeca Diez-AlarciaEmanuele MondaRebecca TallmadgeEmanuele PignattiRebuma FirdessaEmil E. KhavkinReinaldo Molina-RuizEmil MladenovReinhard HehlEmil R. BulatovReinhard KösterEmilia CirilloReka A. HarasztiEmília HanusováRemco T. MolenhuisEmiliano GiardinaRenald DelanoueEmiliano MoriRenata CiccarelliEmilie WidemannRenata FinelliEmilio Mármol-SánchezRenata FreitasEmily GravesRenata Matraszek-GawronEmily GroopmanRenata O. DiasEmily PlaceRenata Rivera-MadridEmily StephensonRenata SlomnickaEmma C. CacciolaRenato De FilippisEmma L. DuncanRené Massimiliano MarsanoEmma Timmins-SchiffmanRengyun LiuEmmanouil TampakakisRenu WickremasingeEmmanuel MosesRenzo MignaniEmmanuelle FabreReuben M BuckleyEmre AksoyRex T. NelsonEnas M. El-BallatReza AkbarzadehEng Guan ChuaRhewter NunesEnoch A. MensahRia CahyaningsihEnrica AlicandriRiadh IlahyEnrico ApaRicardo FonsecaEnrique JaimovichRicardo GunskiEran ElhaikRicardo Jorge LopesErandis Torres-SanchezRicardo LebrónErgun KayaRicardo Sánchez De MadariagaEri KawashitaRicardo SantosEric BatschéRicardo UtsunomiaEric BoncompagniRicardo Villa-BellostaEric De Camargo SmidtRicha SalwanEric OlsonRichard BurchellEric P. SpanaRichard FinnellEric YenRichard J. GibbonsErica SalvatiRichard J. RebelloErik BootRichard John Bruce FrancisErik L. JensenRichard Jude SamulskiErika Di ZazzoRichard Karlsson LinnérErik-Jan KamsteegRichard Kumaran KandasamyErin L. DamsteegtRichard LebaronErnesto PicardiRichard LemalErola Pairo-CastineiraRichard S. JonesErtan Mahir KorkmazRichard SindenEskezeia DessieRick SturmEslam G. El NebrisiRifat Ullah KhanEsmaeil MehraeenRiikka KiveläEspinosa Martín Juan CarlosRik GijsbersEssam SolimanRik SchrijversEstanis NavarroRikky RaiEsther U. KadieneRita GroschEstíbaliz AlegreRita ShiangEugenia CunhaRitesh MenezesEunike VelleuerRitesh ThakareEun-Joo LeeRitu GuptaEunsu ParkRob WhiteEva AltrockRobersy SánchezEva BagyinszkyRobert A. GrahnEva KlopockiRobert AustinEva KriegovaRobert BestEva ŠatovićRobert BroshEva TvrdaRobert E. HutchisonEvangelia ChavdoulaRobert FuentesEvangelia KoutelouRobert H. EiblEvans N. NyabogaRobert HarrisonEvanthia BletsaRobert J. SickoEverlyne M’mbone MulekeRobert KleszczEvert NjomenRobert MorrisEvgenii Germanovich SkurikhinRobert MoyleEvgeniy S. BalakirevRobert OnzimaEvgeny E. AndronovRobert PodlasekEvie MelanitouRobert RyanEwa BalcerczakRobert S. ZemetraEwa Boniewska-BernackaRobert ShenkarEwa KuźnickaRobert ŚmigielEwa Miller-KasprzakRobert Van LooEwelina Elert-DobkowskaRobert W. ReidEwelina PiktelRobert Walter MannEwelina Semik-GurgulRoberta FraschiniEwelina WarzychRoberta MiloneEwelina Warzych-PlejerRoberta RussoEzgi OzkurtRoberto FerrariEzio PortisRoberto PalumbiF. G. Van SteenbeekRoberto PiergentiliFábio A.Abade Dos SantosRoberto SchreiberFabio CianferoniRoberto SorrentinoFabio Matos ChiarelliRoberto Zamora-BustillosFabio MendesRoberto Zenteno-CuevasFabio TimeusRobin DartFabrizio DamianoRobin K. BagleyFabrizio GentileRobin WilganFabrizio MafessoniRocio T. Martinez NunezFabrizio OlivieriRodolfo IulianoFahad Karim KidwaiRodrigo Fortunato De OliveiraFahad KhanRodrigo VidalFahiel CasillasRohan PalmerFaisal RamzanRohit KoloraFan PingRohit ShuklaFan ZhangRohit SrivastavaFangwei ShaoRokayya SamiFanju MengRoland AkakpoFanzani AlessandroRoland JägerFaris F. BrkicRomana VulturarFarzad KianersiRomer RabarijaonaFarzaneh KordbachehRomina RomanielloFatehi FoadRómulo S. SobralFatemeh Kazemi-LomedashtRon KorstanjeFatemeh KhatamiRongfeng CuiFatemeh TabatabaieRoohallah Saberi-RisehFathiya Al MurshediRosa DivellaFatih HanciRosa FregelFatih HatipogluRosa María Giráldez-PérezFatima CvrčkováRosa María Oliart-RosFátima MartelRosario Francisco-VelillaFatma O. KhalilRosario LinaceroFawad AliRoser Gonzàlez-DuarteFederica AlessandriniRoser UrreiztiFederica CirilloRossella CacciolaFederica Di SpiritoRossella TalottaFederica GiuglianoRossiana BojinovaFederica MagagnoliRounak DeyFederico CappellacciRowan MilnerFederico Manuel GiorgiRoy HajjarFederico PlazziRozaihan MansorFederico V. PallardóRozeena ShaikhFei ChenRubén A. FerrerFeiqiao Brian YuRuben C. PetreacaFelice CrocettoRubén CalotoFelicitas PfeiferRuchika DehinwalFelicity VearRudi AppelsFelipe SarmientoRudi VennekensFelix ChanRui ChenFelix HeinrichRuiyang BaoFelix Jimenez-RondanRukang ZhangFemitha PournamiRumesh RanjanFeng ChengRunxiang ZhangFeng GuRuosen XieFeng LiRupert W. StraussFeng WangRupesh DeshmukhFeng XuRupesh Kumar SinghFengjie SunRupesh SrivastavaFengli ZhaoRuslan KalendarFernanda CosmeRussell J. ButterfieldFernanda FortunatoRūta UrbanavičiūtėFernanda NunesRuth TachezyFernández Luís Garcia-MoyaRuxandra-Maria MihaiFernando Augusto Lima MarsonRwik SenFernando M. RohanRyan D. SullivanFernando MarsonRyan LoganFernando Martínez-MorenoRyder OliverFernando Sánchez LasherasRyo MatsushimaFevzi BardakciRyo NishijimaFilipe LiuRyoko Machida-HiranoFilipe PintoRyszard SlezakFilippo De CurtisRyuji HiramatsuFilippo RosselliRyutaro AkiyamaFilomena AdegaRyza A. PriatamaFinn HallböökS. A. I. S. M. GhazaliFiona UkkenS. Chafai ElalaouiFiorella ColasuonnoS. F. EL GioushyFiras KobeissyS. SeemaFirdoos Ahmad GogryS. Yu. FunikovFiroz AkhterSaadet Mercimek-AndrewsFlávio BreseghelloSaba TabasumFleming MargaretSabba MehmoodFlor María Pérez-CampoSabeeta KapoorFlorian KraftSabiha KhatoonFlorian KrompSabina Antonela AntoniuFlorian RaibleSabine Lutz-BonengelFlorin OnisorSabine MaiFlorin TriponSabrina ZidiFlorina MoldovanSabyasachi DashFotis TsetsosSachin NaikFranca AnglaniSachin VermaFranca FagioliSachit AnandFrancesca Anna CupaioliSadaruddin ChacharFrancesca CaponSaddam SaqibFrancesca ClementinaSadia ZafarFrancesca De FalcoSaeng-chuto KepaleeFrancesca DumasSafdar AliFrancesca LonghenaSagar AryaFrancesca M. PirasSagarika MishraFrancesca PalladinoŞahin-Çevik MehtapFrancesca SciontiSahra UygunFrancesco FerriniSaid ShamiehFrancesco M. AngeliciSaif Ur RehmanFrancesco NardiSaiful Effendi SyafruddinFrancesco SalvatoreSairah YousafFrancesco SartiniSairam RudrabhatlaFrancisco Cammarata-ScalisiSajid AliFrancisco CastilloSajid ShokatFrancisco CurateSalesh Kumar JindalFrancisco G. Véliz-DerasSalim MorammaziFrancisco J Sánchez- RiveraSalima Machkour-M’RabeFrancisco J. FernándezSalman WaqasFrancisco J. NavarroSalvatore PastaFrancisco Javier Alvarez-MartinezSalvatore SavastaFrancisco Javier Navas GonzalezSalvatore ScaccoFrancisco TustumiSalvo Danilo LombardoFranco LacconeSamantha GinnFranco MusioSamantha IsenbergFrancois EyskensSamantha KendrickFrank HartungSamar G. ThabetFrank NicholasSambantham ShanmugamFrank R. WendtSambit MishraFransiska MalfaitSameer ChavanFranz KleinSameh AbdelnourFraser CombeSameh ElhadyFrauke StankeSamer ShalabyFrédéric ChainSamia El-MahdyFrédéric G. BrunetSampa GhoseFrédéric Nicolas DaussinSamrita DograFrederic TortSamuel Abalde LagoFrederique PeronnetSamuel Henrique KamphorstFredrik H SterkySamuel KollerFritz RathjenSamuel McLenachanFujie ZhaoSamuele GrecoFulvia FerrazziSamy Mahmoud H. SayedFumikazu MatsuhisaSananda MondalFurhan IqbalSandeep Kumar ChavanFuu-Jen TsaiSandeep Kumar MishraFuxuan WangSandeep SinghG. Ian GallicanoSandipto SarkarG. Suresh KumarSandra BechtelGábor MátyásSandra P. NunesGabor PozsgaiSandra PetrovicGabriel Clemens DworschakSandra RebeloGabriel I. BallesterosSandra Rovira-AmigoGabriela CouxSandrine BarbauxGabriela DumitruSandrine CaburetGabriela Maria CornescuSandrine HouzéGabriela Oana BodeaSandro BanfiGabriela SalinasSang Jin KimGabriela VillanovaSang Won ParkGabriele SaretzkiSanghyun ParkGabriele SicilianoSangita PalGabrielle RudolfSanjay SaxenaGaël RouéSanjiban ChakrabartyGaetano OdiernaSanjit MukherjeeGaetano RiemmaSanthosh ArulGagandeep SagguSanthosh Kumar PasupuletiGaia GoteriSantos AlonsoGail BrionSantos CastañedaGajender AletiSantosh A. MisalGaladriel A. Hovel-MinerSantosh BishnoiGalina DegtjarevaSantosh UpadhyayGalina RalduginaSantosh V. SuryavanshiGanesh C. NikaljeSaqib HassanGanesh UmapathySara A. Abdel GaberGang ChenSara C. ZapicoGang LiSara FaggionGang LiuSara J. HansonGang PengSara MumtazGarry WongSara N. RichterGary S. JohnsonSara Palomo-DíezGastón AlfaroSara Torres RusilloGaurav KandoiSarad Kumar MishraGauri PrasadSarah A. CummingGautham KolluriSarah AshleyGavin McCluskeySarah BajanGayani GalhenaSarah GrovesGeeta RaoSarah HullGeetesh K. MishraSarah K. GilesGeir BjørklundSarah Muniz NardeliGelan AyanaSarahani HarunGeMaria Addolorata MariggiòSaravanan KMGen ZouSaravanan RajuGeng LiSareena SahabGennaro Mariano LenatoSarfaraz ShafiqGenxi ZhangSarfraz ShafiqGeorg RosenbergerSargis KarapetyanGeorg SchlachtenbergerSarvesh Pratap KashyapGeorge DuncanSasivimon SwangpolGeorge FthenakisSatomi KohnoGeorge H. ThompsonSatoshi OkazakiGeorge I LambrouSatoshi YamaguchiGeorge IliakisSatyendra C. TripathiGeorge Konstantinos PapadimasSau W. CheungGeorge P. KostyukSaulo D. PassosGeorge P. LaliotisSaurabh SinghGeorge PopescuSaverio Bartolini LucentiGeorge SakoulasSayaka MiuraGeorge SangsterSayed Haidar Abbas RazaGeorgios AthanasiadisSayyed Ali SamadiGeorgios DiamantopoulosScott E. HickeyGerald BarryScott EdwardsGerald Fritz SchusserScott ReidGerardo Alves Fernandes JúniorSean M. BurgessGerhard HaidlSean Robert AsselinGerhard StegerSebastian Demyda PeyrásGermana MeroniSebastián JaurretcheGetúlio Pereira De OliveiraSebastian RademacherGéza BereckiSebastian RamosGhada Abd-Elmonsef MahmoudSebastian T. BalbachGhada BaraketSebastian ZeidlerGhada El KamahSebastiano A. G. LavaGhazala KaukabSebastiano CiccoGhazala NawazSébastien Sébastien WieckowskiGheona M. AltarescuSeema KumariGholamreza GohariSeiho NagafuchiGiacomo ColtroSelman UluisikGiacomo MorrealeSena ArdicliGianluca Lorenzo PerrucciSenthil Kumar KaruppannanGianluigi MazzoccoliSenthilkumar PalanisamyGil Jae ChoSenthilkumar RajagopalGilbert ErianiSenthilnathan LakshmananGilberto ManzoSeong Hwan ParkGilles KauffensteinSepalika Swarnamali Baththana MudiyanselageGillian I. RiceSepideh Zununi-vahedGilmara Gomes De AssisSerap Özer YamanGioacchin IannoloSerena GasperiniGiorgia BussuSergei I. GrivennikovGiorgina SpecchiaSergei NechaevGiorgio MarozziSergey A. PotapovGiovanna D’EliaSergey FedotovGiovanna DamiaSergey KolomeichukGiovanna D'AndreaSergio Gastón CaspeGiovanni ForcinaSergio Piñeiro-HermidaGiovanni La PennaSergio Rius-PérezGiovanni NigitaSerkan GülGirmantė JurkšienėSethu M. MadhavanGiseli KlassenSeung-Hyun LeeGiulia FassioSeyed Ali HemmatiGiulia FisconSeyed E. HasnainGiulia RioloSeyed Ehsan EndermiGiulia TarquiniSeyed Mohammad Gheibi HayatGiulio FormentiShahid M. BabaGiulio PilusoShahnawaz JadejaGiuseppe BadagliaccaShahram NiknafsGiuseppe Lo BelloShahzad AliGiuseppe SilvestriShailendra GoelGiuseppina De PetroShaimaa SelimGiusy TornilloShajahan AnverGloria González-AseguinolazaShakeel AhmadGloria Yepiz-PlascenciaShakir AliGoichi MiyoshiShane AustinGokce Altay TorunerShane DeneckeGökhan AydoganShang LiGokhan GorgisenShan-Ju YehGopal R. GowaneShannon KinneyGostel MorganShannon PinsonGourav SharmaShaoan WangGoutam DeyShaobo YaoGoutam Kumar TantiShaoni MukhopadhyayGraciela GarcíaShaoping LuGraeme BolgerShaoqing WenGraham M. SnyderSharath Chandra GaddelapatiGrażyna Jarza̧bek-BieleckaSharmila GhoshGrażyna Markiewicz-ŁoskotSharmishtha MusalgaonkarGrażyna SygitowiczSharon SinghGregory BergShaun MasonGregory J. FonsecaShauna Farr-JonesGregory TawaShauna KaplanGregory W. KonatShedrach Benjamin PewanGreig JoilinShefali VermaGrzegorz ChrzanowskiShen Linda GuGrzegorz Jakubiak‪Shengqian XiaGrzegorz JanuszShengyi LiuGuang ShiShensong XieGuanglin HeShenuarin BhuiyanGuangsheng YuanSherif T.S. HassanGuangzhao LiSherine Nagy SalehGudrun A BrockmannShewa BassnetGuido FaviaShiau Foon TeeGuido ZavattaShicheng GuoGuilherme Cordenonsi Da FonsecaShidou ZhaoGuilherme Luis PereiraShigeki ItoGuillaume BanneauShigeru SuzukiGuillaume Butler-LaporteShihlung ChenGuillaume J. Van EysShihua ZhangGuillem Pintos-MorellShihuo LiuGuillermo GiovambattistaShijun LiGulden GokcayShikha Talwar BassiGunārs LācisShilong ChenGunter SchmidtkeShimaa SakrGünther H. S. RichterShimi MeleppattuGuodong WangShimin ZuoGuohao WangShin Ichi ArimuraGuokun YangShing-Hwa LiuGuoyue ChenShin-ichi HorikeGurdeep SinghShinichiro OgawaGurmukteshwar SinghShinji AsanoGurrieri FiorellaShiou Yih LeeGuru MaitiShishir Ram ShettyGustavo Cordero-BuesoShiva Najafi KakavandGustavo De AraújoShiwani SharmaGwendolyn Vaughn ChildsShixi ChenGyan Prakash MishraShixiong ZhangGynendra Kumar RaiShiyan QiaoGyorgy HutvagnerShiyi YinGyörgy László FeketeShogo TsurutaGyörgy SinkovitsShoichiro KokabuH. Rangel-VillalobosShoichiro TangeHab. Tomasz J. PrószyńskiShovan NaskarHadi Pirasteh‑AnoshehShu GuoHafiz Faseeh Ur RehmanShuan-Pei LinHafiz Ishfaq AhmadShubhendu PaleiHaibo LiuShuchen FengHaichao ZhaoShudai LinHaichuan WangShuhei YamadaHaider MejbelShulin YangHaihong ShenShuming LuoHailin WangShung-Te KaoHailing ZongShuo WangHaipeng LiuShu-Pin HuangHaitao WangShu-Ping WuHaitham Abo-Al-ElaShyam Sundar DeyHaiwen LiShyi-jou ChenHaixiao HuSiavash Salek ArdestaniHaiyang LiuSibel DervişHajime MuraguchiSibte HadiHajk-Georg DrostSidharth MehanHakan BozdoğanSigit PrastowoHakim ManghwarSigrid C. RobertsHakim Ouled-HaddouSijung YunHala AbdelmigidSilvia KalantariHala Mounir AghaSilvia LakatošováHala Tabie El-BassyouniSilvia NaitzaHali Librahim OzturkSilvia RocchiccioliHamada ElwanSilvia RussoHamed KavehSilvio SalviHamid BeikiSimon ChangHammad Nadeem TahirSimon John Christoph SoerensenHamsu KadriyanSimón PobleteHamutal MazrierSimon TröderHan MeiSimona BernardiHan Naung TunSimona BoitoHan ZhangSimona CabibHana AlharbiSimona GiglioHana FakhourySimona PagliucaHanaa R. M. AttiaSimona ZaamiHanbing SongSimone BrogiHang SuSimone Di FrancoHanlin XuSimone E. F. GuimarãesHanna FuchsSimone FattoriniHannachi NadjiSimone Reichelt-WurmHannah MaudeSimone ScalabrinHanne IngmerSimone SchmittgenHans G. DrexlerSimran VenkatramanHanyang CaiSimranjeet KaurHao LiSingh RajenderHao YuSinosh SkariyachanHao ZhangSi-Qing LiuHaobin ZhaoSiroj BakoevHaowei DuSirous NaeimiHaq Abdul ShaikSishuo WangHarcharan S. DhaliwalSiva DurairajanHardeep Singh TuliSiyuan SuHardik DesaiSlawomir JarekHardik KundariyaSneha RaoHarri SavilahtiSneha SitaramanHarshpreet ChandokSnehal NirgudeHaruka HandaSnehalata PawarHasan ÖnderSnezhina LazovaHassan RasouliSo MasakiHatem A. ElmezayenSofia Barbosa-GouveiaHazım Serkan TenikecierSohail AhmadHeathcliff Dorado GarcíaSoheil Abbaspour-RavasjaniHeather C. EtcheversSohier YahiaHeather EdgarSohini ChakrabortyHeather JohnsonSoile Jokipii-LukkariHea-Young LeeSok Kuan WongHeba EbeedSolmaz NajafHeba ElsayedSom DevHedia ZitouniSomayeh ReiisiHee Bok ParkSompong ChankaewHee-Geun JoSoňa KřížkováHeejin KimSonali NashineHeidi RoßmannSonghua ShanHeidy M. Marin-CastroSongli ZhuHeike KroegerSongyun LaiHeiko BrennenstuhlSongzhe FuHela Chikh RouhouSonika AhlawatHelen CrookeSoon Ae KimHelen MottSoon KimHelen NigussieSoon Wook KwonHelen R WarrenSoong Deok LeeHelena Correia DiasSophie NicoleHelena MachadoSoren SeifiHélène GaillardSorin MihaliHelga V. TorielloSorin MunteanHelmut SchaschlSorina Mihaela PapucHeloise BastiaanseSouheil GaouarHemant K. MishraSoumya Prasad PandaHenriett ButzSoumyadev SarkarHenry C. ChungSourour AyedHenry D. R. AlbaSouvick RoyHenry S. GibbonsSpencer GibsonHerbst AllenSreelakshmi SreenivasamurthyHernan Garcia-RuizSreyashree BoseHesham A. El-EnshasySrikanth MandaHeshan Sam ZhouSrinivas MummidiHidekatsu YanaiSrinivas R. ViswanathanHidekazu TakahashiSriramajayam KannappanHideki YahirodaSrivatsan RaghunathanHidenori NishiharaStaffan SvärdHidetaka YAMADAStanislav KotlyarovHieu X. CaoStanisław DzimiraHiginio López-SánchezStanley SciortinoHilal Ahmad RatherStavroula MiliHimani PuniaStefan NicolauHin Hark GanStefan PfeifferHind AlsharhanStefan PrekovicHiroetsu SuzukiStefan WinterHirohiko TaniStefania ScaliseHirohisa KishinoStefania VaiHiroki UraStefano CapomaccioHiroki YokotaStefano CastellanaHiroshi AraiStefano Giuseppe CaraffiHiroshi NonoguchiStefano MarziHiroshi SakaueStella Mitrani-RosenbaumHiroyuki ArakiStephan C. CollinsHiroyuki WakiguchiStephan Joel ReshkinHisahide NishioStephan LorenzenHisashi ItoStephan Van Der ZwaardHisato YagiStephan VogtHiu Chuen LokStéphane BoissinotHo-Hyung WooStéphanie ToméHolger BudahnStephen A. RenshawHolli Loomans-KroppStephen LyonHon Cheong SoStephen M. ModellHong Jo LeeStephen PorterHong YangStephen TsoiHong ZhaiSteve WiltonHongbo LiuSteven GreenwayHongfang JinSteven Lim Cho PeiHonghua HuSteven SmithHonglian YeStuart KimHongliang ZhuSu Cheong YeomHong-lin JiangSubhadip ChoudhuriHongquan QuanSudi PatelHongsik KongSue CotterillHong-Tao LiSuganthaPriya ElayapillaiHongwei LiangSuhas Sampat KharatHongyan ShengSuhas V. VasaikarHongyan XuSuhel GaouarHong-Yu ZhangSuhendan EkmekciogluHongzhu CuiSujay GhoshHorst Von BernuthSujit BasakHortensia Moreno-MacíasSujit JagtapHossam RushdiSulagna SaittaHossein TaghizadehSuleiman A. IgdouraHouria DaimiSulev KoksHoussem BoulebdSuman AsallaHoward E. BoudreauSuman ChowdhuryHsiang-Han ChenSumbul SaeedHsiang-Yu LinSuna Onengut-GumuscuHsian-Yu WangSundaravelpandian KalaipandianHsin Hua LeeSung Un HuhHsiuying WangSunghan KimHsueh-Ping ChuSung-Hyen LeeHu GuangweiSungSoon FangHua SunSun-Hyung LimHua YeSunil JaimanHua ZhongSunil RaiHuang LiSunjeet KumarHugues J. ChapSunwoo HanHui ChenSuong Tuyet Thi HaHui LiuSuraiya A. AnsariHui MaoSuravajhala PrashanthHui ZhaoSurendra MeenaHuibin HanSurendra RajpurohitHuihua WangSuresh DamodaranHuinan LiSuresh KalathilHumberto AponteSusan ClarkHumberto GutiérrezSusan DuckettHumberto Sanchez GonzálezSusan ForsburgHuntington PotterSusan RichterHuoqing ZhengSusan WesslerHuria MarnisSusana Amaral TeixeiraHusam QanashSusana BalcellsHussain Yar KhanSusanne DihlmannHussein Mohamed Elhusseiny Ali ElbayoumiSushil DubeyHye-Ryoung KimSushil KumarHyung KimSusumu MitsuyamaHyun-Woo KimSuzanne Ahmad Al-BustanIain WilsonSvein SolbergIan ButtsSven HerzogIan M. CollinsSven WinterIan StanawaySvenja Dannewitz ProssedaIanni AndreaSvetlana A. RomanenkoIbolya AndrásSvetlana CherlinIbomoiye Domor MienyeSvetlana GorokhovaIbrahim Al-AshkarSvetlana GoryunovaI-Ching ChouSvetlana M. MotylevaIda PhillipsSvetlana TamkovichIdit ShacharSwagata Ghatak GhatakIdris LongSwapan ChakrabartyIgnacio PonzoniSwapan Kumar RoyIgnazio La MantiaSwee Lay TheinIgor F. ZhimulëvSwetha RamadesikanIgor I. KireevSyed Farhan AhmadIgor OvtchinnikovSyed Haris OmarIgor RogozinSyed Mudassir JeelaniIgor SplichalSyed Naqui KazimIhab YounisSyed Shams Ul HassanIk-Hwan HanSylvain CarrasIlaria FantasiaSylvaine BoissinotIlaria GrossiSylviane MarouillatIlaria SaltarellaSylvie DiochotIlias SkeparniasSylwia Keller-PrzybyłkowiczIlias TsochantaridisSylwia OkońIlka HeinemannSylwia Olimpia RzońcaIlya Nikolaevich MedvedevSzymon JanczarImmacolata CastellanoT. J. HollingsworthImre HolbTabito KinoImtiaz RandhawaTadeusz PawelczykIn Ja ParkTaemin KimIn Ki ChoTaesic LeeInas S. M. SayedTafun OzcelikInayat Ur-rehmanTaguchi GoroIndika Neluwa-LiyanageTaha Koray ŞahınIndra RamasamyTahir H. SamigullinIndu G. RajapakshaTahsin ShoalaIndu KohaarTaichiro IshigeIndumathi PattaTaiko ToIneke Van Der BurgtTailang YinInese CakstinaTaimoor SheikhIngming ChiuTaisen IguchiInKyeom KimTakahito WadaInkyung JungTakane Kikuchi-UedaInna ChabanTakaoki SaneyasuInna Rabinovich-NikitinTakaomi AraiInna SolyanikovaTakashi OhbaInnocenzo RaineroTakayuki NittaIn-Su ChoiTakehiro IwataIoan HutuTakeshi KawakamiIoana C. RotarTakeshi YaoiIoana CrisanTakiy BerrandouIoana GrozeaTaku OkamotoIoana StreațăTakumi IshizukaIoannis A. GiantsisTakuro TobinaIoannis IliopoulosTakuya KoieIoannis VamvakarisTakwa GabrIpsita MohantyTalita Sarah MazzoniIra A. HerniterTamar KrugmanIram LiaqatTamara Nikuseva MarticIrena NalepaTamás Gergely MolnárIrene BottilloTamer AlshafieIrene MadrigalTanel PungaIrene NetchineTanguy FenouilIrene Vázquez-DomínguezTania CrisanIrfan S. KathiriyaTanja P. VasićIrina BakloushinskayaTanveer SinghIrina ElchevaTanvi SinhaIrina I. MohorianuTao LinIrina MateiTao XuIrina St. LouisTao YangIrina TicaTao ZhangIris BiebachTaobo HuIris Boraschi-DiazTara CarthyIrving Cancino-MuñozTara SudhadeviIsaac BragaTariq MukhtarIsabel MarquesTariq PervaizIsabel VeladaTasiu IsahIsabelle Brun-HeathTatenda GocheIsabelle PerraultTatiana DeniskovaIsabelle ThiffaultTatiana GolubevaIsao IshiiTatiana KostrominovaIshara S. ManawasingheTatiana MaroilleyIshtiaq AhmedTatiana SimaoIsidoro FelicielloTatiana V. ShelengaIskander IbrahimTatiane FernandesIslam RafiadTatijana ZemunikIsmael BoussaidTatsuhiko GotoIsrael Enrique Padilla-GuerreroTatsuya KobayashiIsrar AhmadTed BrownIstvan NagyTe-Hua HsuIstván NagyTejas BosamiaItay BarneaTemidayo ElufisanItzi Fragoso-MartinezTemidayo S. OmolaoyeIulia MateiTeng ZhouIva MozgovaTeodora PapovaIvan Delgado EncisoTeresa Donze-ReinerIvan LandiresTeresa GasullIvan MalčićTeresa J. Donze-ReinerIvan NombelaTeresa LettiniIvan RaveraTeresa Rubio-TomásIvan S. PetrushinTeresa Tórtola FernandezIvan SabolTeresa ŻołądekIvana BilicTerje RaudseppIvana DelalleTerry A. WheelerIvana FridrichovaTerry Ann ElseIvana NovakovicTertia Purves-Tyson Purves-TysonIvona Djurkin KušecTeruaki TozakiIwona GłażewskaTeruyuki MatsunagaIwona RzeszutekTetsuo KuniedaIyappan RamachandiranTetyana ZayatsIzabel Cristina Rodrigues Da SilvaTeweldemedhn Gebretinsae HailuIzabela Szućko-KociubaThalida E. ArpawongIzhar Hyder QaziThangavelu MuthukumarIzzat TahirThankappan RadhakrishnanJ. C. Luis JorgeTheivanayagam MaharajanJ. Francis BorgioTheivasigamani ParthasarathiJ. Peter W. YoungThemis GiannoulisJ. Spencer JohnstonTheo KantidakisJ. Stephen C SmithTheodora PapamitsouJaakko L. O. PohjoismäkiTheodore P. BraunJaan ViidaleppTheodoros KarantanosJaana LahdetieTheoni MargaritopoulouJacek GawrońskiTheresa A. SpradlingJacek KamczycTheresa StrongJack D. SunterThilini WijesekeraJackeline Rossetti MateusThippa Reddy GadekalluJack-Yves DeschampsThiruppathi Senthil KumarJacob EllegoodThiruvenkadan Aranganoor KannanJacob KageyThomas BartnikasJacqueline HarrisThomas BlankersJadranka VranekovićThomas CaspariJadwiga ŻebrowskaThomas HollemannJae Hoon BahnThomas LiehrJae-Won ChoiThomas LopdellJaganmay SarkarThomas LuxJakub CieślakThomas MohrJakub RokThomas NavesJakub SawickiThomas SmolJakub VašekThomas SvobodaJamal NaderiThomas Van Overeem HansenJamal NasirThomas VogtJames B. GibsonThomas WernerJames D. LordThorsten BraunJames DavisonThorsten SchinkeJames DemastesThroe EgelandJames JancovichTiago Benedito Dos SantosJames MickelsonTiago OlivotoJames SaccoTian LiangJames Van EttenTianpeng ZhangJamshad HussainTien-Yuan WuJan BocianowskiTiffany M. DelhommeJan Ching Chun HuTija C. JacobJan ŠevčíkTilen ZamljenJana MoravcikovaTilman Sanchez-ElsnerJana SchäferTimm KonoldJana ŽiarovskáTimofey RozhdestvenskyJanakiram R. VangalaTina B. McKayJanani ParameswaranTina DidariJanay Almeida Dos Santos SerejoTina ParadzikJanet DavisTing ZhangJared NordmanTingming LiangJarosław BurczykTingting DongJasenka WagnerTingting YangJasmine W. TayTingyu ChangJason FlannickTinyiko Edward HalimaniJaume GardelaTiong Yang TanJaved Mehedi ShamratTodd BilleJavier CanalesTodd HsuJavier Perez-GarciaTodd MichaelJavier Pizarro-CerdaTohru FujiwaraJavier PlasenciaTomás NaranjoJavier Rodriguez-CentenoTomasz BlicharskiJaviera Ortiz-SeverínTomasz GambinJawad KhanTomasz LitwinJay JayaramanTomasz PłoszajJay V. PatankarTomasz SadkowskiJayanta DasTomasz Za̧bekJayasankaran ChandruTomer VenturaJean René HuynhTomislav TostiJeanaflor Crystal ConcepcionTommaso NicolettiJean-Baptiste Le PichonTomoki HoshinoJean-Francois GrossetTomoko UeharaJean-François SoucyTomomi YamaguchiJeanine AmacherTomoyoshi KomiyamaJean-Marie ExbrayatTong LiuJean-Pierre GagnerTope OyeladeJean-Yves RoignantTorben RedmerJeffery A. DahlbergToru YagoJeffrey BlanchardToshihiro NomuraJeffrey L. WoodheadToshko LjubomirovJeffrey M. KiddTosso LeebJeffrey M. LiptonTran Dang KhanhJeffrey RogersTrine B. OpstadJei-Wan LeeTristan BouschetJe-Keun RheeTsing BohuJelena P. Jovičić-PetrovićTsutomu ShimuraJelena RamljakTsz Kin Martin TsuiJelica Grujić-MilanovićTuan XuJennifer Ellen PoseyTulin ArasogluJennifer J. JohnstonTusar Kanti BeheraJennifer L. MacNicolTuyen T. DangJennifer ThomsonTze-Kiong ERJennifer ZitserUday Chand JhaJen-Tsung ChenUehara TomokoJeong-An GimUfuk İlgenJeremy R. EgbertUgur Cengiz ErismisJeremy TimmisUğur ŞenJeremy WiluszUlhas Sopanrao KadamJérôme D. RobinUlises De La Cruz-MossoJerzy GrabińskiUlisses Moreno-CelisJes-Niels BoeckelUlrich KellnerJessica HekmanUlrike BaranyiJessica J. WangUlrike MietzschJessica Sheu-GruttadauriaUma ChandrachudJessy A. SlotaUmar ZebJesús Fernández-LucasUmashankar ChandrasekaranJia Xian LawUmberto MalapelleJiakun YanUmesh GoutamJialei DuanUmmara WaheedJiali YuUri HamielJian FengUri NirJian LiUrminder SinghJian LinUsama Abd El-RazekJian ShiUwe WalldorfJian WangV. Mohan Murali AcharyJian ZhangV.G. AbilashJian ZhaoVadim LebedevJianbo WangVagner Ricardo LungeJianbo ZhangVaibhav ChandJianfang WangVaibhav UpadhyayJianfeng MeiVaidutis KučinskasJian-Fu ZhangVaishnavi MuralikrishnanJiang ZhongValentina CuriniJiangqi WenValentina GinevičienėJianhai ChenValentina NaefJianhui XieValentina PirotaJianing FuValentino BezzerriJianJun JinValentino GiarolaJianli DongValeria De PasqualeJianli TaoValeria MarigoJianling XieValeria OrlandoJianlong XuValerie A. ArboledaJianlou NiuValerio CaputoJiannan ZhangValerio RicciJianping ZhangVanesa Santás-MiguelJianxin JiangVanessa De SantisJianye GeVanja Mandic-MaravicJiawei WangVarun BansalJiaxu LiVasfiye Kader-EsenJiayu XueVasia S. MavrogianniJicheng ZhaoVasilia FasoulaJichun ChenVasiliki KaravaJie LiVasiliy ChokheliJie YangVasily SukhorukovJie ZhaoVasily V. StavchanskyJie ZhuVassiliki I. EvangelouJifang ZhangVassiliki StamatopoulouJignesh H. KamdarVassilios DotasJihad OrabiVasudevan RamachandranJiHan XiaVeeranoot NissapatornJim MyersVelimir MladenovJim SheuVenera FerritoJimena GuerreroVenkata ChalamJin ChenVenkata Rajesh YellaJin LiuVenura HerathJin ZhangVenusia CortelliniJinchen LiVeronica De PaolisJindrich CitekVerónica F. Morán-BarrosoJing ChenVeronica MartiniJing HeVeronica NobileJing PangVeronika Hulejová SládkovičováJing TangVeronika R. KharzinovaJing WeiVesna M. CoricJing YangVicente Eliezer Vega-MurilloJing ZhangVictor A. Palomino-TapiaJingang XiaoVictor G. LevitskyJingjing XuVictor HernandezJingwen CaiVíctor Hugo Pérez-EspañaJingyu FanVictor M. PulgarJinhong KanVictor TreviñoJin-Liang ZhaoVictor Van BeusechemJintao WangVictoria ShneyerJinu HanVictoria SleightJiří BonaventuraVidmantas BendokasJiri NeuzilVidya SagarJiunn- Liang KoVieira Társis PaivaJiun-Wen GuoVijay IndukuriJiwen WangVijay PandeyJo SourbronVijay Rakesh Reddy SanikommuJoachim GeyerVijaya Anand ArumugamJoana GonçalvesVijayachitra ModhukurJoana R. LoureiroVikas KumarJoanna BogusławskaVikas Kumar RoyJoanna Drozd-SokołowskaVikas SharmaJoanna GruszczyńskaViktor ČernýJoanna KaminskaViktória KoroknaiJoanna KuliszVilda PurutçuoğluJoanna Matowicka-KarnaVinay Kumar TyagiJoanna Rzeszowska-WolnyVinaya Kumar KatneniJoanna SajewiczVinayak M. JoshiJoanna StojakVincent HoJoanna SzczepanekVincent P. DiegoJoanna Wessely-SzponderVincent PonsJoanna ZarzynskaVincent ReyesJo-Anne BrightVincent-Philippe LavalléeJoão Carlos Caetano SimõesVincenzo AuriliaJoão Farias GuerreiroVincenzo La BellaJoão GonçalvesVincenzo ZappavignaJoao GuilhermeVinesh VinayachandranJoão José GouveiaViney GuptaJoão MachadoVinit ShanbhagJoão Renato SebastiãoVinod JakkulaJodi Woan Fei LawVinod TiwariJoel EllwangerVioleta G. TruscaJoerg-Ingolf SteinVioleta PopoviciJohannes HoferVirginia Herrera-ValenciaJohannes K. EhingerVisanu WanchaiJohn BeverleyVishnu TripathiJohn D. HamillVishnukiran ThuragaJohn FyfeVitaly KozinJohn GatesyVito BiondiJohn H. BoyleVittoria DisciglioJohn Jay GargusVivek BhardwajJohn KyndtVivek YadavJohn LaiVivekanand TiwariJohn MaciejowskiViviana GenualdoJohn Paul PlazzerVlad DimaJohn S. BuckletonVlad GorduzaJohn S. ChoyVlad PădureanuJohn SorensenVladan BogdanovićJohn Timothy W. WrightVladimir A. LukhtanovJohn W SteeleVladimir VladimirovJolanta Behnke-BorowczykVladimir YeremeevJolanta ChmielowiecVladislava GusarJolanta RzymowskaVlatka Cubric-CurikJonas CicenasVolker EnzmannJonathan ColemanVolker MohlerJonathan GresselVrinda S. ThakerJonathan NeilsonVui King Vincent-ChongJonathan PetersVyacheslav YurchenkoJonathan R. SkinnerWafik ZakyJonathan Robert MaceyWahab A. KhanJongmin KimWahyu KusumaJoon-Yong AnWaill ElkhateebJoost BaertWajid ZamanJorddy CruzWaldemar WagnerJordi PijuanWalter MossJorge ChiapellaWang HaifengJorge Diogo Da SilvaWankun XieJorge Oscar ChiapellaWansheng LiuJorge Paredes MonteroWanting HanJose Angel FontenlaWanying WuJosé Antonio Fernández-RobledoWaqar MajeedJosé Antonio Garrido-CárdenasWaqar ShafqatJosé Cleiton Sousa Dos SantosWardatou BoukariJosé Daniel CaminoWaseem El-HuneidiJosé Felipe Warmling SprícigoWaseem HassanJosé Francisco IslasWasim AhmadJosé Luis Pérez-CastrillónWassim AlmawiJose PuzoWayne PowellJose Sanchez-BetancourtWedad S. SarawiJosé SuazoWei Ching KhorJosee Guirouilh-BarbatWei GanJosef FinstererWei LiuJosef Jr. FulkaWei YanJoseph P. DewulfWei-Chieh LeeJoshua B. GrossWeidong BaoJoshua K. MeisnerWeihong QiJoy MitraWei-Hsien ChienJoyce Ching Mei LamWeihua HuangJoydeep MitraWeijia SuJozsef VighWeikai YanJP CamachoWeilai DongJrhau LungWeiren LuoJuan C. OliverosWeisheng CaoJuan C. Ortiz-MarquezWeitao LiJuan Carlos Del PozoWeiyan JiaJuan Carlos LacalWeizhen LiuJuan Carlos ZentenoWen ChenJuan E. RosasWenbin ZhangJuan Enrique Rodríguez-PérezWenchao XuJuan GómezWenfeng GuoJuan M. Guillen-MuñozWeng Kung PengJuan Manuel Guzmán-FloresWenge LiuJuan-Francisco Peña-CardellesWenhua ZhangJudit García VilloriaWenjiang DengJudit OláhWenjin ZhengJudith L. RoeWen-Jun GaoJudith LópezWenliang XuJudy SavigeWen-Lii HuangJuergen ReichardtWenqiang SunJuhi Raju UttamaniWenqing JiangJulia M. WanguiWen-Sheng LiuJulia MetzgerWentao DongJulia O. FedotovaWenting LiJulia TcwWenyan XuJulián L. Zayas-ArrabaWieland HermannJulian Nevado BlancoWieslawa LeśniakJulian RozenbergWilbert P. VermeijJuliana Amaka UgwuWill NashJuliana Maria Navia-PelaezWilliam A. CantaraJuliana PetriniWilliam Adam GowerJuliana Silva BernardesWilliam B. MillerJulie PhillipsWilliam Bart BryantJulien Van GilsWilliam C. MerrickJulio AguadoWilliam CamuJulio Augusto Freyre-GonzalezWilliam DuddyJúlio César De SouzaWilliam Earl FryJulio Cesar Dos ReisWilliam GrafJulio Plaza DíazWilliam Kirby GottschalkJulius Pyton SserumagaWindy McNerneyJulius RablWing-Tat PoonJun AkibaWladimir Bocca Vieira De Rezende PintoJun HeWladimir MauhinJun HirayamaWojciech BielskiJun MaWojciech KozdruńJun RenWojciech SzlasaJun TabataWojciech WitarskiJun TangWolfram KunzJun WangWolyna PindiJun YaoWon Byoung ChaeJun ZhouWoojin KangJun ZhuWoolfolk SandraJunaid AhmedWoori KimJunaidi KhotibWorapong SingchatJune SimpsonXavier Bofill-de RosJung Sun KimXavier RamboutJung SunyoonXavier RocaJunhong ZhangXenia LatypovaJunhui PengXi LouJunko TakahashiXi ZhangJunliang YinXian LuoJunmin PanXiang WangJunna HeXiang ZhouJunya KobayashiXiangpeng YueJuraj MedoXiangshu DongJürgen SchmitzXianguang GuoJustin TackneyXianjin QiuJustyna Rewak-SoroczyńskaXianren JiangJuulita KulbackaXianyong YinJuvenal Rodríguez-ReséndizXianzhi ZhangJyothi BadriXiao LinK. Ann HorsburghXiao ZhangK. H. Katie ChanXiaodong WangK. L. RavikumarXiaoen HuangK. V. RavishankarXiaofei ChenKaalak ReddyXiaogai HouKabiru AkinyemiXiao-Hong LuKaela VarbergXiaohua DaiKaewmongkol GunnXiaoju HuKai HuXiaokang WuKai KammersXiaolei ZhuKai LuoXiaoli HuKai WangXiaoLong KangKai WilleXiaolu WeiKai YuXiaoning ZhangKaihui LuXiaopeng TangKaio AleviXiaoshu XuKaisa IvaskaXiaowei ZhangKaiyang ZhangXiaoxu LiKalaiselvi SenthilXiaoyan DingKalaivani ParamasivanXiaoyu LiKallayanee ChawengsaksophakXiaoyu ZhangKalra Rajkumar SinghXin BiKalyani DhusiaXin ChenKamalakar ChatlaXin LinKamaleldin SaidXin TongKamel A. Abd-ElsalamXin WangKamel Abd-ElsalamXin’e ShiKamil Mert EryalçınXinan Holly YangKamila Kusz-ZamelczykXingchen PengKanat GürünXinhui WangKang HeXinlian ShenKangjin JeongXinlu DingKannan SridharanXinru WangKannan Vrindavan ManianXinxu YuanKanut LaoharaweeXiujun ZhangKara CervenyXiuling LiKarel Jan AngelisXiurong YangKaren M. HoughtonXiurong ZhangKaren UsdinXu ChenKarim Ben M'BarekXueming ZhaoKarine Assis CostaXueqin GaoKarine DubranaXuewei YangKarishma ChhabriaXuewu DuanKarl B. AndreeXuexian ZhangKarl ClarkXuexiu ZhengKarl Martin KleinXuezhao LiuKarl-Philipp RommelXuling ChangKarolin HeinzeXuqiao ChenKarolina BoguszewskaYaddanapudi RavindranathKasia BebenekYafei DuanKatalin KomlosiYaguang ZhangKatarina KouterYahaya TajudeenKatarina LukšićYakov DemurinKatarína RažnáYakup KaskaKatarina VukojevicYali SunKatarzyna KodziszewskaYamei LiuKatarzyna KorybalskaYan CuiKatarzyna KoziakYan GuoKatarzyna KrawczykYanan WangKatarzyna LubeckaYanbo FanKatarzyna M. TycYang LiKatarzyna PiórkowskaYang MingKatarzyna Ropka-MolikYangyang SongKatarzyna SikorskaYangyong LvKatarzyna WigluszYanjun GuoKatarzyna ZaorskaYanjun KouKate E. LynchYanjun LiKate KellerYanning ZuoKate TsaiYaohua YangKate YoungYapeng SuKaterina KatsarakiYaping TianKaterina TrejbalovaYaqiang CaoKaterina-Marina PilalaYash GuptaKatharina HöferYashika RustagiKatharina SchimmelYasufumi OmoriKatherina WalzYasuhisa OhataKatherine E. OdegaardYasuhito IshigakiKathleen ClaesYasuko IshidaKathleen MorrillYasunari MatsuzakaKathleen NichollsYasuyuki YamadaKathleen V. AxenYavar VafaeeKathrin MarheinekeYavuz Selim YıldırımKathryn LeyvaYaw AniwehKatie L. AyersYawei LiKatie L. PenningtonYayoi IzuKatja Helena RönkäYe LiuKatja OdeningYen-Liang LiuKatrin DomschYeyang FanKatrin VogtYheni DwiningsihKatrine M. JohannesenYi ChenKavindra Kumar KesariYi LeeKavitha S. RaoYi LiaoKazi Ahsan HabibYi WangKazuhito ToyookaYi XiaoKazunori ShimizuYiao WangKazuo YudohYi-Ching LeeKazuya ShiratoYi-Chung LeeKazuya YamagataYifeng QiKe XiaoYih-Fung ChenKee Siang LimYijing ZhouKeerthie DissanayakeYi-Ming ChenKees NoordamYin QiKeiko MiyoshiYing ShenKeira J. A. JohnstonYing ShengKeisuke KozaiYing WangKeitaro KanieYing YanKemal Melik TaşkınYinghui LiKemal Tuna OlğaçYingying YeKen TakasawaYino WuKeng Ioi VongYinwen ZhangKenji IkeharaYi-Qing YangKenneth K. KiddYiwang ZhouKenneth McElreaveyYixuan CaoKenneth VernickYixuan MaKensuke KumeYogesh Kumar VashistKerstin BystrickyYogesh VikalKesawat Mahipal SinghYolanda GogorcenaKeshav GopalYolande SaabKessen PattenYong ChengKeting ChenYong-Bi FuKevin B. MyantYongfang XieKevin ChengYonggang LuKevin GlintonYong-Gu ChoKewei CaiYonghua QinKhac-Minh ThaiYongjin ParkKhadija BatoolYongjun WeiKhadija El HadriYongning XinKhaled AmiriYongshuang XiaoKhaled ElsayadYongzhen HuangKhaled SalemYoon Young KimKhaleel Ibrahem JawasrehYordan MuhovskiKhalid M MahroseYorihiro IwasakiKhalil KhamassiYork MarahrensKhamis YoussefYoshihiro HottaKhan BushraYoshihiro IshikawaKhurram LiaqatYoshihisa MatsumotoKhurshid AhmadYoshikazu OhyaKhushdeep BandeshYoshimitsu FujiiKi Wha ChungYoshinobu IchimuraKibrom B. AbrehaYoshio KatoKieran G. MeadeYoshiya IkawaKi-Kwang OhYosuke ToyodaKim KeelingYounes Rezaee DaneshKimberly RowlandYoung Eun YoonKinga GawelYoung-Kyo SeoKinga HadzsievYoung-Rog YeoungKinga Humińska-LisowskaYoung-zoon KimKingsley EkwemalorYoussef KhamisKira BehrensYu AnKira V. VyatkinaYu GaoKira ZadesenetsYu GeKiran UpadhyayYu H. SunKirill AntonetsYu SunKirill UstyantsevYu XuKirk GastonYu YangKirk StephensonYu. D. NechipurenkoKirsten A. CoprenYuan LiuKirstin JanssenYuan LyuKirthiram K. SivakumarYuan YuanKi-Tae HaYuan ZhouKiyoshi EgawaYuanchun WangKlára KosováYuanfeng ZhangKlaudia KruppaYuanqing YanKlaudia PawlinaYubang ShenKlaudia Pawlina-TyszkoYubbu PutriKlaus H. HoffmannYubin MaKoichiro WasanoYuchen LiuKoji MikamiYu-Chia HsuKok-Min SeowYuehuan ZhangKomal RamaniYue-Lin ZhuKomiljon TojibaevYueqiao HuangKomuraiah MyakalaYueqiu TanKonda Mani SaravananYufang YinKongju ZhuYugo AshinoKongpan LiYuichiro TanakaKonosuke NakajiYuji YasukochiKonrad CelińskiYujun ZhuKonrad HuppiYuk Fai LeungKonstantin KhrapkoYuki NagamatsuKonstantinos ArsenopoulosYuki TabataKonstantinos KoudounasYukio NaganoKonstantinos SousounisYuko HaradaKonstantinos VoskaridesYuko KatoKookhui RyuYulia GrishchukKorakrit ImwattanaYulong SunKorkut UlucanYun Kit YeohKoshi AkahaneYun MaKostas IoannidisYunhao WangKosuke KataokaYunhui PengKotb AttiaYuntao HuKoteswararao GarikapatiYunus Emre BeyhanKouichi KawamuraYun‐Xia LuanKouichiro KawanoYunxiang ZangKourosh VahdatiYunxiao ZhangKoushiro OhtsuboYunyan ShengKrinio GiannikouYuri BozziKris ChristensenYuri GogolevKrishna YadavYuri Lvovich OrlovKrishnamoorthy SrikanthYurij BukinKristaps JurjansYuriy L OrlovKristiaan Van Der GaagYuriy Lvovich OrlovKristian Karsten UllrichYuriy OrlovKristian T. SchafernakYury LoikaKristine Barlow-StewartYusen ZhouKristine KrajnakYusuf OladosuKrisztián FrankYusuf SertKrisztina BelaYusuke MurakamiKrystyna CwiklinskiYutao LiuKrystyna Mazan-MamczarzYu-Wei WuKrzysztof AndresYuxiang LiKrzysztof KowalYuyoung JooKrzysztof KrystaYves SabbaghKrzysztof LewandowskiYvonne G. WeberKrzysztof TupikowskiZachary D. WallenKuang-Chi ChenZachary GerringKuang-Hung ChengZahid Hameed SiddiquiKubilay YıldırımZahid MahmoodKui ZhangZahra AnvarKuldeep GuptaZahra BeyzaeiKumar Gupta SandeepZakaria KehelKumi YoshidaZakariya AlgamalKun XiaZan LiKunihiro TsukasakiZarrin BasharatKunka Mohanram RamkumarZeeshan AhmadKunqi ChenZehuan LiaoKuntal DeZeineen MominKuppusamy SivasankaranŽeljana BašićKurt ReinhartŽeljana PrijićKurubaran GanasegeranŽeljko AntićKushagra BansalŽeljko ReinerKwan-sik MinŽelko JelečKwanuk LeeZenon LukaszewskiKyle BurghardtZeyan LiKyle SueZhaofu YangKyoko YamaneZhaolong YuKyoungYul SeoZhaozhong ZhuL. M. SureshZhe JiL. Maximilian BujaZhe WangL. Morrison ClaireZhe ZhangLada Lukić-BilelaZhen HuangLaetitia MaugeZhen LuLahiru HandunnetthiZheng ChenLai WangZheng FuLaia Rodriguez-RevengaZheng WangLaima BalčiauskienėZhenghan WangLakmini SenavirathnaZhengxing LianLan XiongZhenhui ZhongLanre MorenikejiZhenlin LiLara-Flores MiguelZhenxin FanLarisa AnghelZhenya TangLarry CroftZhenyong DuLars Joachim LindbergZhenzhen XuLászló SzemethyZheyang WuLata I. ShuklaZhi ZhengLata SinghZhi ZouLaura AudìZhibin LvLaura CorbinZhibo MaLaura E. GarciaZhicheng LinLaura EllestadZhigang LiLaura García-CorzoZhihai LiuLaura García-PosadasZhijie QiLaura LapagliaZhijun DaiLaura M. SackZhijun GuoLaura TomaselloZhili ZhengLauren Appleby GayZhiqiang HuLaurence DrouilhetZhiying DengLaurent CalvierZhi-Ying WuLaurent MullerZhiyong LiuLauria MassimilianoZhiyuan YangLavinia CabaZhongfeng YeLavinia RechZhonghou TangLaxman KhanalZhonghou WangLe LuoZhonghua LiuLeander CorrieZhongming ZhaoLeandro Exequiel LuceroZhongshi ZhouLeandro SantiagoZhongtian LiuLee Bulla JrZhongwei LiLee Hwan-youngZhuangwei ShiLei HeZhuanjian LiLei WanZiguo ZhuLei ZhangZilhas Ahmed JewelLei ZhouZiliang LuoLeif AnderssonZiqing ZhaoLeif KirsebomZirui DongLeigh Anne ClarkZisis KozlakidisLeila GasmiZissis MamurisLeila JahangiriZitao ChenLeila RiahiZoha KibarLeili Saeednejad ZanjaniZohaib KhurshidLei-Shih ChenZoltán IvicsLejla PojskicZoltan MagyarLenka KlčováZongzhao ZhaiLeny C. GalvezZsolt DemetrovicsLeonardo BascoZurisaday Santos-JimenezLeonardo Emberti GialloretiZuzana VivodováLeonardo Murgiano



